# Isolation, structural characterization, immunomodulatory and indirect tumoricidal activities of an inulin-type fructan from *Platycodon grandiflorus* via macrophage M1 polarization through the TLR4/NF-κB pathway

**DOI:** 10.1007/s13659-026-00657-5

**Published:** 2026-11-02

**Authors:** Chen-Xi Wang, Ping-Yan Liao, Cui-Lian Yin, Yue Wang, Jie Hu, Kui Li, Zhao-Kun Li, Yu-Jie Sun, Xian-Yang Li, Jiang-Miao Hu, Ning Li, Meng-Ting Kuang

**Affiliations:** 1 Anhui Key Laboratory of Bioactivity of Natural Products, School of Pharmacy, Anhui Medical University, Hefei, 230032 China; 2 The Tenth Clinical Medical College of Guangzhou, University of Traditional Chinese Medicine, Zhongshan, 528400 China; 3 Zhongshan Hospital of Traditional Chinese Medicine, Zhongshan, 528400 China; 4 State Key Laboratory of Phytochemistry and Natural Medicines, Kunming Institute of Botany, Chinese Academy of Sciences, Kunming, 650201 China

**Keywords:** *Platycodon grandiflorus*, Macrophage M1 polarization, Indirect tumoricidal activity, Immunomodulator, TLR4/NF-κB pathway

## Abstract

In this study, a homogeneous polysaccharide, designated PGP-1, was successfully isolated and purified from the roots of *Platycodon grandiflorus*. Comprehensive structural characterization via methylation analysis, GC–MS, and NMR spectroscopy identified PGP-1 as an inulin-type fructan predominantly composed of →1)-β-D-Fru*f*-(2→ as the main chain with terminal β-D-Fru*f*-(2→ and α-D-Glc*p*-(1→ residues, consistent with the structural features of previously reported *Platycodon grandiflorus* fructans. Unlike conventional chemotherapeutic agents that rely on broad-spectrum and non-specific cytotoxicity, biological evaluations revealed that PGP-1 acts as a safe and targeted immunomodulator. Specifically, PGP-1 effectively induced M1 macrophage polarization in RAW264.7 cells through the TLR4/NF-κB signaling pathway, significantly enhancing their phagocytic capacity and promoting the robust release of tumoricidal factors, including nitric oxide (NO), TNF-α, and IL-6. Crucially, utilizing a macrophage-tumor transwell co-culture model to simulate the tumor microenvironment, it was demonstrated that PGP-1 exhibited negligible direct cytotoxicity to cancer cells but exerted potent indirect tumoricidal activity against hepatocellular (H22) and lung (LLC) carcinoma cells by augmenting macrophage-mediated immune responses. These findings provide preliminary evidence linking the polysaccharide's safety profile with its tumor-suppressive efficacy, suggesting the potential of PGP-1 as a low-toxicity natural immune enhancer for cancer immunotherapy.

**Graphical Abstract:**

**Figure:**
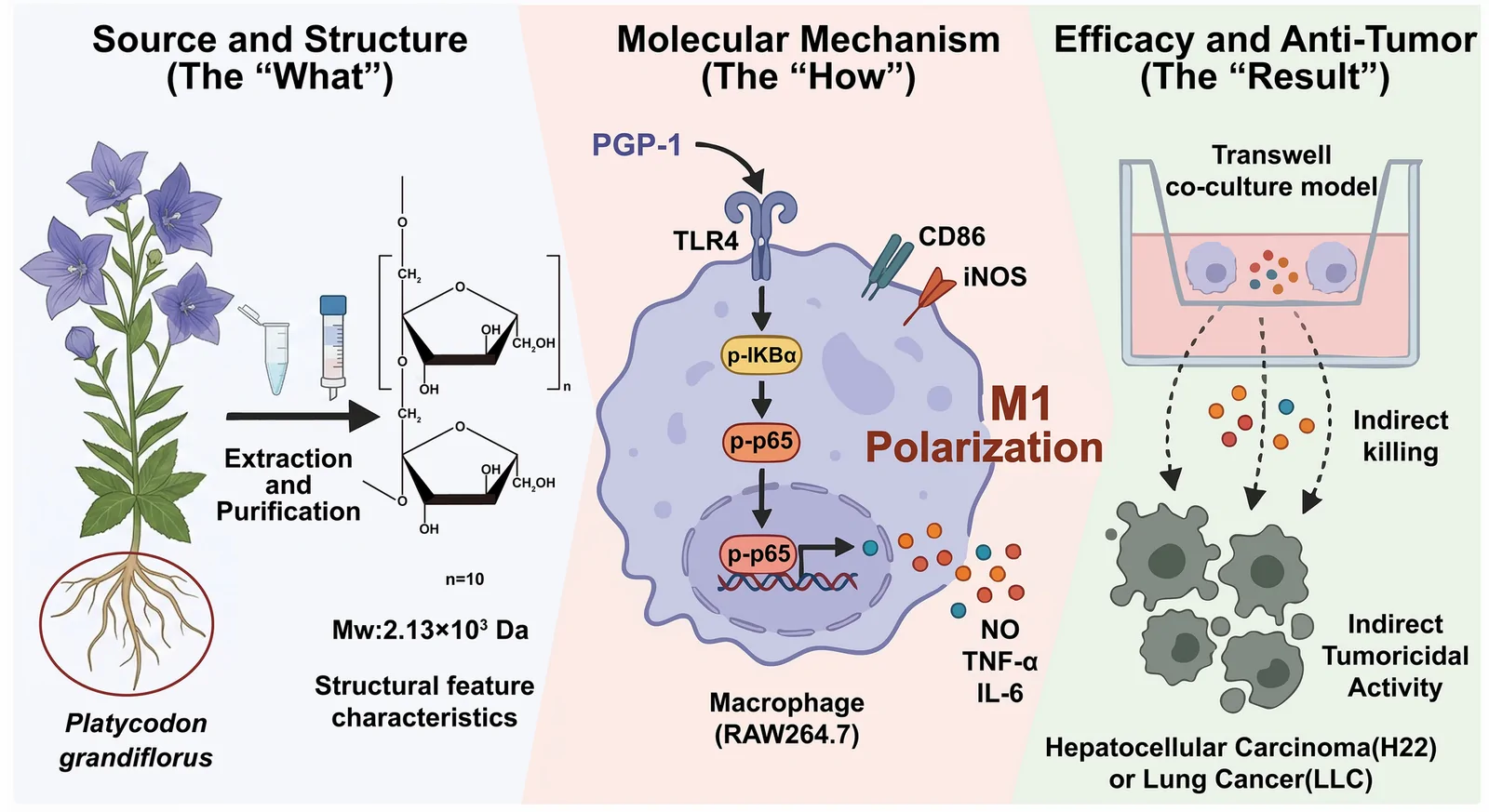

**Supplementary Information:**

The online version contains supplementary material available at https://doi.org/10.1007/s13659-026-00657-5.

## Highlights

- A homogeneous inulin-type fructan (PGP-1, Mw 2.13 × 10³ Da) was purified from *Platycodon grandiflorus* and structurally characterized by 2D NMR.
- PGP-1 selectively induces M1 macrophage polarization via TLR4/NF-κB without affecting M2 markers.
- PGP-1 inhibits tumor cells indirectly through macrophage-mediated tumoricidal activity in co-culture models.
- First study linking safety and tumoricidal efficacy of a *P. grandiflorus* fructan via M1 polarization.

**Supplementary Information:**

The online version contains supplementary material available at https://doi.org/10.1007/s13659-026-00657-5.

## Introduction

The tumor microenvironment (TME), particularly its immune cell composition and polarization, critically influences tumor progression and therapy response [1, 2]. While immune cells like macrophages represent promising therapeutic targets in the TME [2, 3], a major bottleneck in current cancer therapy is the severe adverse effects caused by conventional strategies [4–7]. Unlike conventional chemotherapeutic agents that exhibit direct and broad-spectrum cytotoxicity to both malignant and normal somatic cells, naturally derived bioactive macromolecules, particularly plant polysaccharides, offer a safer and highly promising paradigm. They function as precise immunomodulators rather than direct cellular poisons, effectively remodeling the immunosuppressive tumor microenvironment with significantly lower toxicity.

*Platycodon grandiflorus* (Campanulaceae), a valued medicinal plant and functional food ingredient in China [8], exhibits diverse health benefits, including anti-inflammatory, antioxidant, and immunomodulatory effects [9–11]. Polysaccharides, as the key bioactive constituents in *Platycodon grandiflorus*, demonstrate stimulating immune factors (TNF-α, IL-1β, IL-6, IFN-γ) and inhibiting tumor cell viability (e.g., HepG2, A549) [12–15]. However, *Platycodon grandiflorus* polysaccharides are typically characterized as non-toxic and biocompatible macromolecules. A critical scientific issue that remains insufficiently explored in current research is the mechanistic integration of their safety profile with their potent anti-tumor activity. Attributing the anti-tumor efficacy of these macromolecules solely to direct cytotoxicity is inconsistent with their inherent safety characteristics. Therefore, elucidating whether these polysaccharides inhibit tumor growth through direct action or indirect immune mobilization is essential for a profound understanding of their true mechanisms. Within this immune-mediated anti-tumor paradigm, the functional status of key immune cells in the TME serves as a primary research focus. As representative functional cells in the TME, macrophages exhibit high plasticity and play a central regulatory role, with the capacity to be polarized into either the anti-tumor (M1) or pro-tumor (M2) phenotypes in response to extracellular signals [16]. This capacity for phenotypic transition renders macrophages an optimal logical node connecting biosafety with tumoricidal activity, whereby robust host immune responses are induced through mild signaling stimuli. Consequently, the targeted induction of M1 polarization is not only a significant immunotherapeutic strategy but also an ideal pathway to investigate the indirect tumoricidal mechanisms of polysaccharides. While crude *Platycodon grandiflorus* polysaccharides (PGPs) show indications in immunoregulation, the homogeneous polysaccharides remain largely unexplored in their structures responsible for immunomodulatory activities, and precise mechanisms, particularly concerning macrophage polarization.

Based on this background, structural characteristics and immunomodulatory mechanisms of a homogeneous polysaccharide from *Platycodon grandiflorus*, named PGP-1, were explored herein. Although inulin-type fructans from *P. grandiflorus* have been previously reported with prebiotic and immunomodulatory activities [17, 18], their role in the tumor microenvironment, particularly the capacity to mediate indirect tumoricidal effects through macrophage M1 polarization, has never been investigated. Crucially, our observations revealed that PGP-1 exhibited negligible direct cytotoxicity against cancer cells even at a concentration of 200 μg/mL for 48 h. This experimental evidence not only confirms its safety as a plant-derived substance but also supports the premise that its tumoricidal efficacy does not derive from direct cellular damage. Building upon this, this present study aims to elucidate the relationship between safety and tumor suppression: PGP-1 functions as an immune trigger rather than a cytotoxic agent, inducing RAW264.7 macrophage polarization toward the anti-tumor M1 phenotype via the TLR4/NF-κB pathway. In an in vitro co-culture system, we further demonstrated the capacity of PGP-1 to integrate immune promotion with potent tumoricidal activity. These findings collectively suggest that PGP-1 exerts significant tumoricidal effects through the activation of macrophage-mediated immune responses, providing a scientific basis for its potential application as a high-efficiency and low-toxicity natural immune enhancer.

## Materials and methods

### Chemicals and reagents

This specimen (*Platycodon grandiflorus*) was purchased from Kangmei Pharmaceutical Co Ltd, originated from Bozhou, Anhui, China. Standard molecular weight glucans and standard monosaccharides were bought from the National Institutes for Food and Drug Control. DEAE Cellulose-52 and Sephadex G-50 were acquired from GE Healthcare Biosciences Co. Dimethyl sulfoxide (DMSO) and trifluoroacetic acid (TFA) were acquired from Shanghai Aladdin Biochemical Technology Co Ltd. Dulbecco's Modified Eagle Medium (DMEM), phosphate-buffered saline (PBS) and fetal bovine serum (FBS) were from Life Technologies Co. 2-(2-Methoxy-4-nitrophenyl)-3-(4-nitrophenyl)-5-(2,4-disulfonatophenyl)-2H-tetrazolium sodium salt (CCK8) was purchased from APExBIO Technology LLC. SP600125 (1,9-Pyrazoloanthrone), NSC118218 (Fludarabine), BAY11-7082 ((E)-3-Tosylacrylonitrile), TAK-242 (Resatorvid), and NSC15139 (10-Hydroxydecanoic acid) were purchased from APExBIO Technology LLC. Anti-phospho-p65 (82,335-1-RR) were from Proteintech Group, Inc. Anti-iNOS (HA722031), anti-CD86 (ET1606-50), anti-TLR4 (RT1666), anti-p65 (ET1603-12), anti-IKBα (ET1603-6), anti-phospho- IKBα (ET1609-78) were from Huaan Biotechnology Co Ltd.

### Extraction and purification of PGPs

The pretreated powder of *Platycodon grandiflorus* was extracted using hot water. To streamline the manuscript and emphasize the structural characterization and pharmacological mechanisms, the detailed extraction optimization process using single-factor tests and Box-Behnken response surface methodology (RSM) for model fitting has been documented in the Supplementary Materials (Fig. S1, Tables S1–S3). Based on this optimization, the optimal extraction parameters were determined as: heating time of 100 min, heating temperature of 75 °C, liquid-material ratio of 15 mL/g, and two successive extractions. Under these conditions, the average extraction rate of crude PGPs was 18.58%.

Crude PGPs were dissolved in distilled water, and proteins were eliminated using the Sevag reagent (n-butanol: chloroform = 1: 4). Residual organic solvents were removed using a rotary evaporator. This procedure was repeated three times. Finally, the deproteinized PGPs were obtained through lyophilization. The protein-depleted PGPs fraction was dissolved in deionized water (15 mg/mL) and subjected to anion-exchange chromatography using a DEAE-cellulose 52 column. A dialysis bag with a 1000 Da molecular weight cutoff was used to dialyze. After dialysis, the samples were further purified by elution with distilled water on a Sephadex G-50 column. The eluted fractions were analyzed by HPLC-ELSD (high-performance liquid chromatography with evaporative light scattering detection), revealing a single homogeneous peak (PGP-1).

### Structural characterization of PGP-1

#### Molecular weight determination

The MW of PGP-1 was analyzed by a Shimadzu LC-20A instrument equipped with a Shimadzu ELSD-16. A Shodex Ohpak SB-803 HQ column was used, 20 µL aliquots of PGP-1 solution (2 mg/mL) were chromatographically analyzed to establish retention parameters. A calibration curve was generated using dextran standards (D0-D5), enabling computation of PGP-1's molecular weight through retention time correlation.

#### Chemical and monosaccharide composition analysis

Quantification of soluble sugars was performed using the established phenol–sulfuric acid method with spectrophotometric detection [19]. The Bradford method was used to assess protein content [20]. 3 M TFA (1 mL) was used to hydrolyze 2 mg PGP-1, followed by HPAEC-PAD to determine the monosaccharide composition [21].

#### Fourier-transform infrared spectroscopy analyses

A FT-IR spectrometer with a scanning range of 4000–400 cm^−1^ was used to detect the functional groups of PGP-1 (1 mg), which had been crushed and compressed with 100 mg of KBr powder.

#### Methylation analysis

The methylated polysaccharide sample was made in anhydrous DMSO with CH_3_I and NaH. Following acid hydrolysis with TFA, the sample underwent sequential derivatization steps involving sodium borohydride reduction followed by pyridine/acetic anhydride acetylation. The derivatized products were subsequently analyzed by gas chromatography-mass spectrometry (GC–MS).

#### NMR analysis

NMR (Bruker) was used to obtain structural information about PGP-1. Repeat the process of dissolving PGP-1 in 0.5 mL of deuterium oxide three times followed by freeze-dried before transferring the sample to an NMR tube. Subsequently, ^1^H NMR, ^13^C NMR, DEPT-135 NMR, COSY, HMBC, HSQC, ROESY, and TOCSY were performed at 25 ℃.

#### Scanning electron microscope (SEM) analysis

Lyophilized PGP-1 sample (2 mg) was put on a specimen holder and gold was sprayed on it. The sample was observed under a cryo-scanning electron microscope (Sigma 300, Germany).

### Cells and cell culture

Mouse lewis lung carcinoma cells (LLC), mouse hepatocellular carcinoma cells (H22), and mouse monocyte macrophage cells (RAW264.7) were acquired from the Cell Bank of the Chinese Academy of Sciences and China Centre for Type Culture Collection. All cell cultures were incubated at 37 °C in a 5% CO_2_ atmosphere. DMEM supplemented with 10% FBS served as the growth medium for all cell lines used in this study.

### Cell viability assay

The viability of RAW264.7, H22, and LLC cells was ascertained using the CCK8 assay. Briefly, cells were inoculated into 96-well plates for 24 h. The supernatant was then replaced with FBS-free medium containing different concentrations of PGP-1 (or conditioned medium for specific assays). After RAW264.7 cells were incubated for 48 h (and tumor cells accordingly), FBS-free medium and CCK8 (10: 1) were added to each well in place of the supernatant. After 1 h of incubation, absorbance values were quantified at 450 nm wavelength with a spectrophotometric plate reader. The control group consisted of untreated samples (0 μg/mL PGP-1).

### Western blot analysis

SDS-PAGE gels were used to separate equal volumes of denatured total cell lysates after cell lysis. The electrophoresed proteins were moved to a polyvinylidene difluoride membrane by electrical conversion. After 20 min of room temperature blocking with quick blocking solution, the membrane was incubated with the primary antibody for the entire night at 4 °C. The membrane underwent three 10-min TBST washes before and after secondary antibody incubation (1:2000, Epizyme Biomedical), and finally the ECL detection kit (GlpBio) was used to visualize the blots. Western blotting data were quantified using ImageJ.

### RNA isolation and quantitative real-time PCR

RAW264.7 cells treated with PGP-1 or LPS were subjected to RNA extraction using an RNA extraction kit, and the quality and quantity of RNA were measured using a UV–visible spectrophotometer. Under the action of reverse transcriptase, the obtained RNA was reverse-transcribed to synthesize cDNA, which was then used as a template to amplify the target gene using a fluorescence quantitative PCR instrument, and the relative expression of the target gene was measured using SYBR Green Supermix. Primers are listed in Table S4.

### Immunity factor assay

RAW264.7 cells were seeded in 96-well plates (1 × 10^4^ cells/well) and stimulated with PGP-1 for 48 h. Immunological factors released by RAW264.7 macrophages were detected using the corresponding kits according to the instructions. (the NO kit (Beyotime, S0021M), ELISA kit (Multi Sciences Biotech, EK282, EK206)).

### Endotoxin detection and polymyxin B neutralization assay

To exclude the possibility that the immunomodulatory activity of PGP-1 was attributable to endotoxin (LPS) contamination, endotoxin levels in the purified PGP-1 samples were determined using a gel-clot Limulus Amebocyte Lysate (LAL) assay kit (C0266DS, Beyotime, sensitivity: 0.125 EU/mL) according to the manufacturer's instructions. PGP-1 was dissolved in endotoxin-free water at concentrations of 1 mg/mL and 200 μg/mL (the maximum working concentration used in this study). A spike recovery test was simultaneously performed by adding LPS standard (0.25 EU/mL) to PGP-1 samples to verify the absence of matrix interference. After mixing with LAL reagent and incubation at 37 °C for 60 min, gel formation was assessed by inverting the reaction tubes.

Additionally, polymyxin B (PMB) (Y261125, Beyotime) sulfate (30 μg/mL), a specific LPS-neutralizing agent that binds to the lipid A moiety of LPS, was used to further confirm that the immunostimulatory activity of PGP-1 was intrinsic to the polysaccharide. RAW264.7 cells (1 × 10^4^ cells/well) were preincubated with PMB in serum-free DMEM for 1 h prior to stimulation with PGP-1 (100 and 200 μg/mL) or LPS (1 μg/mL, as a positive control for PMB efficacy) for 48 h. NO production in the culture supernatants was measured as described in Sect. 2.8.

### Flow cytometry to detect M1 macrophage-related markers

In order to detect macrophage polarization, RAW264.7 cells were inoculated into 24-well plates (6 × 10^4^ cells/well) for 24 h, followed by 48 h of treatment with PGP-1 (200 μg/mL). After 10 min of CD16/CD32 antibody staining, the suspended cells were subjected to 30 min of surface-labelled antibody staining. The stained cells were then detected using flow cytometry (Beckman CytoFLEX). APC anti-mouse CD86, Brilliant Violet 421TM anti-mouse CD206, PE-Cyanine7 anti-Nos2 (iNOS), PE anti-mouse Arginase 1 antibodies were bought from BioLegend. CD16/CD32 monoclonal antibodies were bought from eBioscience.

### Conditional medium and co-culture assay

RAW264.7 cells were seeded in 96-well plates at a concentration of 2 × 10^4^ cells/well in the conditioned culture system, and the cells were stimulated with PGP-1 (0 μg/mL, 50 μg/mL, 100 μg/mL, and 200 μg/mL) for 48 h, after which the supernatant was collected and noted as conditional medium. H22 cells and LLC cells were inoculated at 1 × 10^4^ cells/well in 96-well culture plates for 24 h and then cultured in conditioned medium for 48 h. Tumor cell viability was determined by CCK8. In the co-culture system, H22 cells and LLC cells were inoculated at 6 × 10^4^ cells/well in 24-well plates for 24 h. Subsequently, inserts containing RAW264.7 cells were placed directly on the top of 24-well plates containing H22 cells and LLC cells. PGP-1 (0 μg/mL and 200 μg/mL) was added to the top inserts and incubated for 48 h. Tumor cell viability was determined by CCK8, and NO, IL-6, and TNF-α levels in RAW264.7 cells were detected.

### Intracellular signaling pathways involved in PGP-1-induced macrophage M1 polarization

To determine the signaling pathways involved in the polarization of macrophage M1 caused by PGP-1, SP600125 (Jun inhibitor), NSC118218 (STAT1 inhibitor), BAY11-7082 (NF-κB inhibitor), TAK-242 (TLR-4 inhibitor) and NSC15139 (IRF-1 inhibitor) were used. In brief, cells were preincubated in DMEM that included one of the following inhibitors 30 μM SP600125, 100 μM NSC118218, 5 μM BAY11-7082 for 1 h [22], 55 μM TAK-242 and 5 mM NSC15139 [23] for 30 min and then exposed to polysaccharides. Measurement of NO secretion as described above.

### Statistical analysis

All the experiments were performed with replicates. ANOVA and the unpaired t-test were used to assess the data for statistical significance, and expressed as mean value ± standard deviation (SD). The level of statistical significance was determined using the following criteria: *p* < 0.05, *p* < 0.01, *p* < 0.001, and *p* < 0.0001. *p* < 0.05 was considered as significant, *p* < 0.01 was considered as very significant.

## Results

### Optimization of PGPs extraction parameters

As noted in the Methods section, the results of the single-factor test and model fitting are detailed entirely in the Supplementary Materials (Supplementary Fig. S1, Tables S1-S3).

#### Response surface analysis

**Fig. 1:**
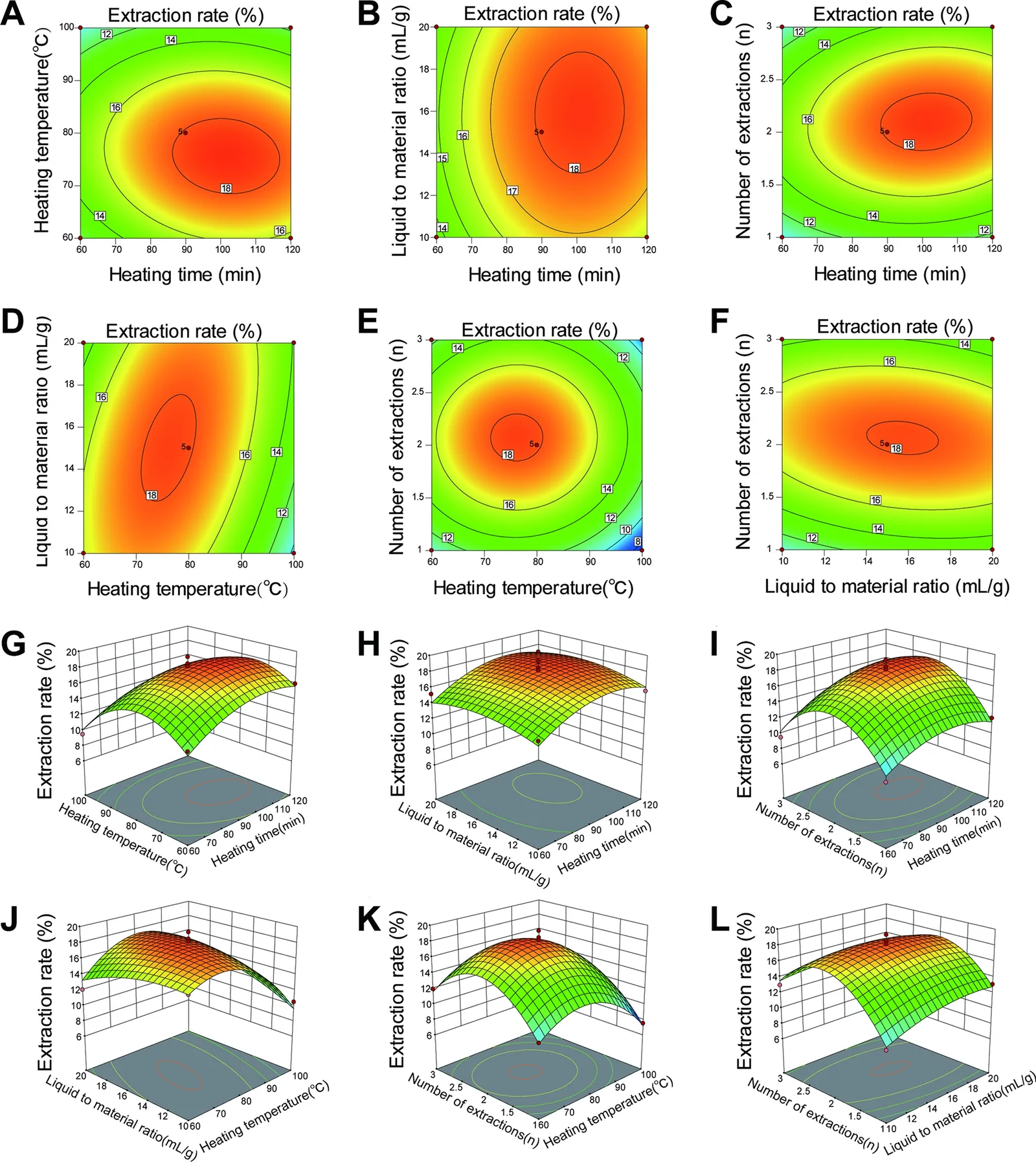
2D contours and 3D response surface plots. **A**–**F** The 2D contour plots of heating time, heating temperature, liquid/material ratio and number of extractions for PGPs extraction yield. **G**–**L** The three-dimensional (3D) response surface plots of heating time, heating temperature, liquid/material ratio and number of extractions for PGPs extraction yield

3D response surface plots and 2D contours generated from the quadratic polynomial model of PGPs extracted yields were used to represent the relationship between the independent variables. The 2D contour plots were shown in Fig. 1A–F, and the 3D response surface plots were shown in Fig. 1G–L, one variable was ignored in one 3D response surface plot and only two of the variables were depicted.

The interactions of heating time with heating temperature, liquid-material ratio and number of extractions were shown in Fig. 1G–I, respectively. The yield of PGPs increased with increasing heating time, peaking at 90 min and then slowly decreasing. The degree of concavity of the 3D response surface plots showed that the interaction of heating time with heating temperature and number of extractions was more pronounced, whereas the extraction rate of PGPs changed only slightly with the increase of liquid-material ratio. Figure 1J, L showed the interaction of the liquid-material ratio with the heating temperature and the number of extractions, and the extraction rate of PGPs changed significantly with the increase of both temperature and number of extractions, while the response curve of the liquid-material ratio was relatively smooth. Figure 1K showed the effect of heating temperature and number of extractions on PGPs yield, and their contour plots were close to circles, indicating that the interaction between heating temperature and number of extractions was not significant, but their respective effects on PGPs extraction rate were significant. Taken together, the extraction rate of PGPs was sensitive to the variation of extraction temperature and insensitive to the liquid-material ratio, which was consistent with the results of the statistical analyses (Table S3).

#### Optimal conditions of PGPs extraction and validation of the model

The response values of the PGPs extraction with the selected conditions were predicted by the generated model equations and the optimal conditions obtained were: heating time of 102.004 min, heating temperature of 75.4 °C, liquid/material ratio of 14.734 mL/g, and the number of extractions of 2.103 times. The predicted maximum response was 18.528%. Validation experiments were then carried out using the adjusted conditions: heating time of 100 min, heating temperature of 75 °C, liquid-material ratio of 15 mL/g, and number of extractions of two. The average PGPs extraction rate determined in five replicate experiments was 18.581 ± 0.010%. The experimental values were not significantly different from the predicted values (*p* > 0.05), which indicated that the optimization of the PGPs extraction scheme by hot water leaching using the established polynomial equations and response surface model was accurate and satisfactory. The high extraction rate of PGPs enabled the present study to focus predominantly on the structural characteristics and precise immunomodulatory tumoricidal mechanisms of the purified active macromolecule, PGP-1. In addition, compared with previous reports on PGPs, not only the response surface analysis of PGPs extraction conditions was performed in this study, but also the developed hot water immersion method has the advantage of less time (100 min vs. 180 min) [15] and energy (75 °C vs. 90 °C) [17] requirements.

### Structural characterization of PGP-1

#### Molecular weight, chemical composition and monosaccharide composition of PGP-1

**Fig. 2:**
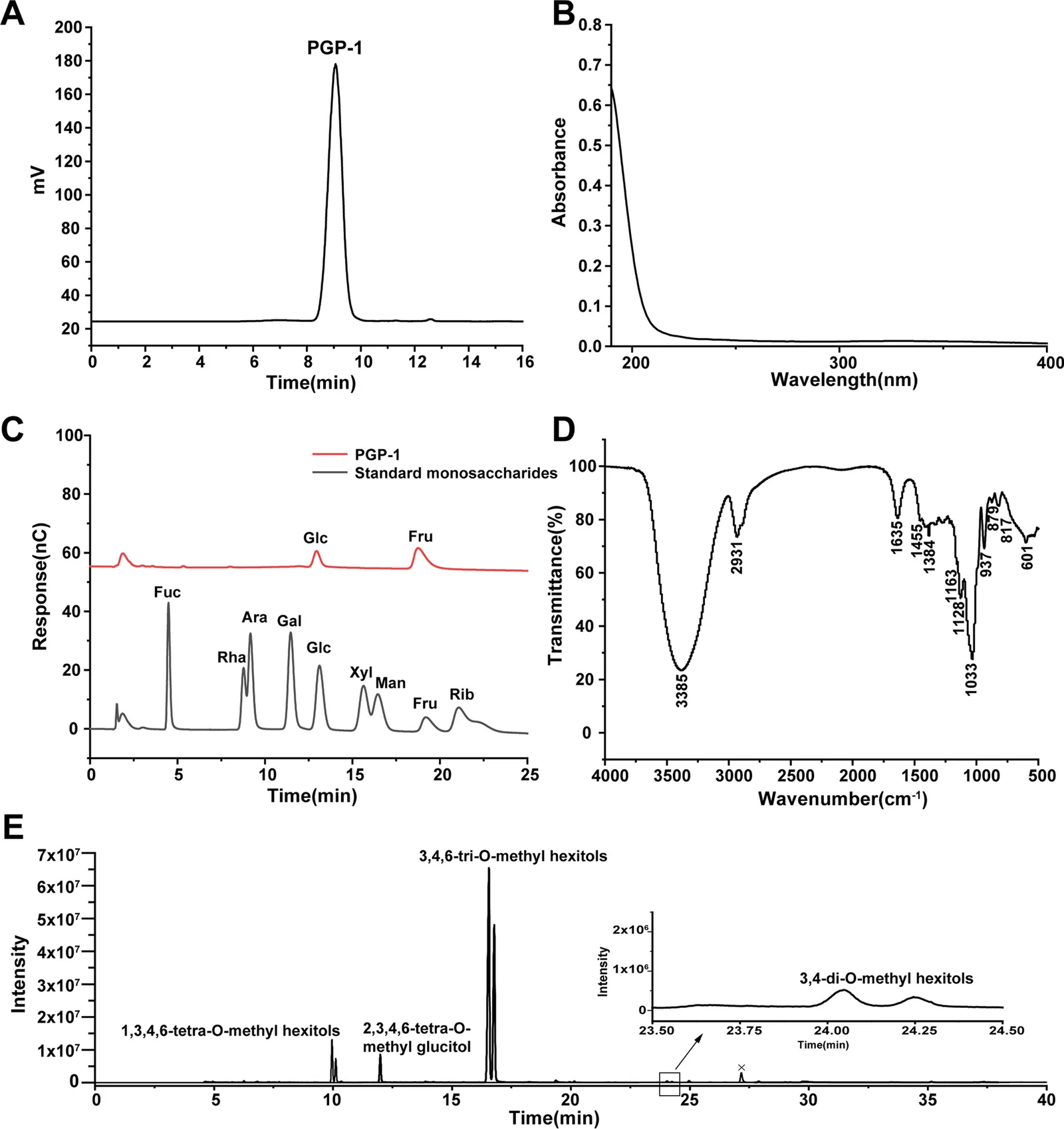
**A**, **B** The HPGPC (**A**) and UV spectrum (**B**) of PGP-1. **C** The monosaccharide composition analysis of PGP-1. **D** IR profiles of PGP-1. **E** GC–MS profile of PGP-1. The minor unidentified peak (marked with ×) does not correspond to any known PMAA

As shown in Fig. 2A, the HPGPC curve of PGP-1 exhibited a narrow symmetrical peak, indicating that PGP-1 is a homogeneous polysaccharide. Based on the calibration curve, the molecular weight was calculated to be 2.13 × 10^3^ Da. The protein and glucuronic acid content of PGP-1 were undetectable, furthermore, the UV spectrum (Fig. 2B) showed no discernible absorption peaks, indicating the absence of protein in the PGP-1 samples. The sugar content was 92.80%. As shown in Fig. 2C, fructose and glucose in a ratio of 90.43:9.57 were confirmed as the monosaccharide component of PGP-1.

#### IR spectral analysis of PGP-1

The FT-IR spectrum of PGP-1 (Fig. 2D) exhibited a pronounced and broad absorption band at 3385 cm^−1^, indicative of O–H stretching vibrations in the sugar residues [24]. Additionally, the C-H bending and stretching vibrations of the sugar ring were observed at about 2931 cm^−1^ and 1455 cm^−1^, respectively [25]. The absorption peak at 1635 cm^−1^ corresponded to the stretching vibration band of C=O. The characteristic signals of the polysaccharides were represented by small band peaks at 1320–1220 cm^−1^, which were also signals of the C-O variable angle vibrations in the sugar ring [26]. The peaks within the range of 1000–800 cm^−1^ were indicative of furanosyl residues. The bands at 937, 879, and 817 cm^−1^ signify the presence of fructose with β-configuration glycosidic linkages in PGP-1 [27, 28].

#### Methylation analysis of PGP-1

GC–MS was used to analyze the partially methylated alditol acetates (PMAAs) derived from PGP-1. Four types of PMAAs were identified in the total ion chromatogram (Fig. 2E), including 1,3,4,6-tetra-O-methyl hexitols, 3,4,6-tri-O-methyl hexitols, 3,4-di-O-methyl hexitols and 2,3,4,6-tetra-O-methyl glucitol. The identity of each PMAA was confirmed by its characteristic EI-MS fragmentation pattern (Table S5 and Fig. S5A-E). It should be noted that each fructose-derived PMAA produced two adjacent chromatographic peaks due to the generation of two diastereomeric alditols (mannitol and glucitol) upon reduction of the 2-keto group, which is characteristic of ketose-derived PMAAs [29]. Accordingly, four linkages were identified as terminal-Fru*f* (t-Fru*f*), →1)-Fru*f*-(2→ (1,2-Fru*f*), →1,6)-Fru*f*-(2→ (1,2,6-Fru*f*) and terminal-Glc*p* (t-Glc*p*), with peak area ratios of 10.91:81.97:2.19:4.93. The predominant linkage was →1)-Fru*f*-(2→, accounting for 81.97% of all sugar residues, indicating that PGP-1 is an inulin-type fructan. The →1,6)-Fru*f*-(2→ linkage was detected only as a trace component (2.19%). Since methylation analysis is inherently semi-quantitative due to the differential molar response factors among different PMAAs [29], the peak area ratios were not directly converted to molar proportions. The discrepancies among the methylation peak area ratios, monosaccharide composition, and molecular weight are discussed in Sect. 4.

#### NMR analysis of PGP-1

The structure of PGP-1 was further investigated by 1D and 2D NMR spectroscopy. Given that methylation analysis identified →1)-β-D-Fru*f*-(2→ as the overwhelmingly predominant residue (82%), the NMR spectra were accordingly dominated by signals from this major repeating unit, with only minor contributions from the terminal residues.

**Fig. 3:**
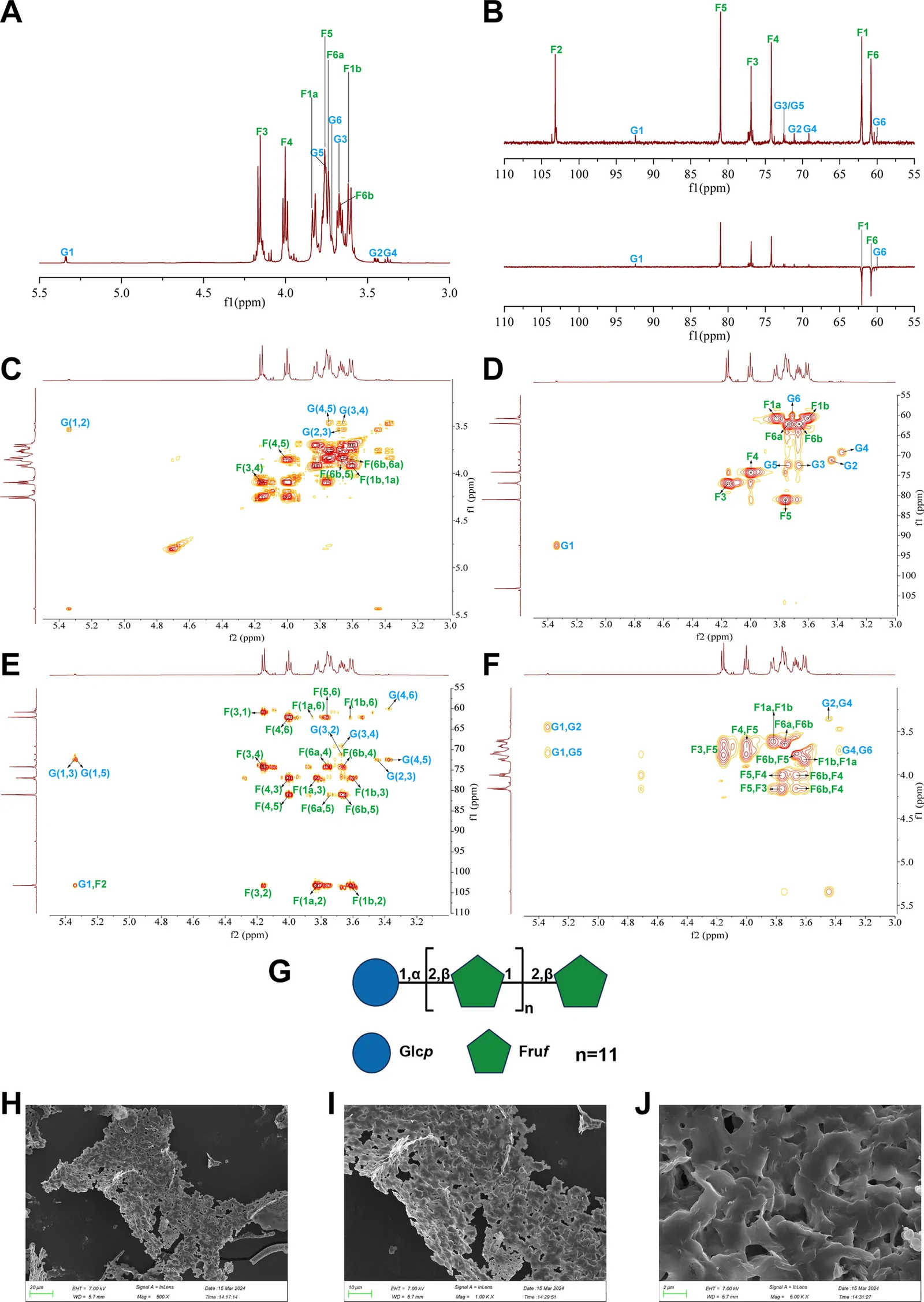
**A**–**F** NMR spectra of PGP-1. ^1^H NMR spectrum (**A**), ^13^C NMR spectrum and DEPT-135 spectrum (**B**), ^1^H-^1^H COSY spectrum (**C**), HSQC spectrum (**D**), HMBC spectrum (**E**), ROESY spectrum (**F**). **G** Proposed structure of PGP-1. **H**–**J** SEM images of PGP-1 in different magnifications. 500 × (**H**), 1000 × (**I**), and 5000 × (**J**)

The ^1^H NMR spectrum of PGP-1 (Fig. 3A) was dominated by a set of intense signals in the upfield region (*δ*_H_ < 4.5), characteristic of fructofuranosyl residues [30]. Since fructose is a 2-ketose bearing a quaternary anomeric carbon (C-2), no anomeric proton signal was observed for the fructose component [31]. A weak but clearly discernible signal at *δ*_H_ 5.34, exhibiting a doublet with a coupling constant of *J* = 3.78 Hz, was detected in the anomeric region and assigned to H-1 of a terminal α-D-glucopyranose residue (Residue G) [32]. The small coupling constant is characteristic of an α-anomeric configuration. In the ^13^C NMR spectrum (Fig. 3B), the anomeric region displayed a strong signal at 103.18 ppm, assigned to C-2 of the β-D-fructofuranosyl repeating unit (Residue F), alongside a signal at 92.42 ppm corresponding to C-1 of the terminal α-D-Glc*p* (Residue G) [33]. Additional minor signals at 103.03 and 103.64 ppm were also observed in the C-2 region, attributable to the terminal β-D-Fru*f*-(2→ and the →1)-β-D-Fru*f*-(2→ residue adjacent to the terminal α-D-Glc*p*, respectively, which reside in subtly different electronic environments compared to the internal repeating units. The DEPT-135 spectrum (Fig. 3B) confirmed the assignment of methylene carbons (CH_2_) at approximately 60–63 ppm, which appeared as inverted signals, corresponding to C-1 and C-6 of fructose residues and C-6 of glucose residues, readily distinguishable from the methine (CH) and quaternary carbons [30].

A comprehensive set of 2D NMR experiments, including ^1^H-^1^H COSY (Fig. 3C), HSQC (Fig. 3D), HMBC (Fig. 3E), and ROESY (Fig. 3F), was employed to achieve detailed chemical shift assignments for the two principal residues. The complete assignments are summarized in Table 1.

**Table 1:** Summary of ^1^H and ^13^C NMR chemical shift of PGP-1

Code	Glycosyl residues	Chemical shifts (ppm)					
		H1/C1	H2/C2	H3/C3	H4/C4	H5/C5	H6/C6
F	→1)-β-D-Fru*f*-(2→	3.84,3.62/60.82	–/103.18	4.16/76.90 (d)	4.00/74.20 (t)	3.76/81.01	3.74,3.66/62.06
G	α-D-Glc*p*-(1→	5.34/92.42 (d, 3.78 Hz)	3.45/71.12 (dd)	3.67/72.54	3.38/69.15 (t)	3.75/72.38	3.72/60.04

For residue F (→ 1)-β-D-Fru*f*-(2 →), the HSQC spectrum provided direct one-bond C–H correlations. Since both C-1 and C-6 of fructofuranose are exocyclic methylene carbons (-CH_2_-O), each bearing two diastereotopic protons that are non-equivalent due to the adjacent chiral centers, they appear as pairs of HSQC cross-peaks in the 60–63 ppm region [31]. No cross-peak was observed at the C-2 position (103.18 ppm) in the HSQC spectrum, verifying the quaternary nature of this anomeric carbon. Starting from H-3 (*δ*_H_ 4.16, d), the ^1^H-^1^H COSY spectrum (Fig. 3C) revealed sequential scalar connectivities, H-3/H-4 (*δ*_H_ 4.00, t) and H-4/H-5 (*δ*_H_ 3.76). The corresponding carbon chemical shifts were determined from HSQC as C-3 (76.90 ppm), C-4 (74.20 ppm), and C-5 (81.01 ppm) [30]. The distinction between C-1/H-1 and C-6/H-6 was established by ^1^H-^1^H COSY connectivity. The proton pair at *δ*_H_ 3.66 showed a COSY correlation with H-5 (*δ*_H_ 3.76), identifying them as the geminal H-6 protons. In contrast, the proton pair at *δ*_H_ 3.84 and 3.62 showed no COSY correlation with any ring proton, consistent with their assignment as the geminal H-1 protons, which are isolated from the fructofuranose spin system by the quaternary C-2 [31]. The glycosylation at O-1 resulted in a modest downfield shift of C-1 (60.82 ppm) compared to the terminal fructose C-1 signal (approximately 60.4 ppm). The observation that C-6 (62.06 ppm) nonetheless resonates downfield of C-1 is a consistent feature observed across reported inulin-type fructans [34, 35], which may be attributed to the combined electronic effects of dual glycosylation at both the O-1 and O-2 positions within the polymer chain [35].

For residue G (α-D-Glc*p*-(1 →), the anomeric cross-peak at *δ*_H_ 5.34/*δ*_C_ 92.42 in HSQC was characteristic of an α-linked glucopyranose [32, 33]. Sequential proton assignments were established via ^1^H-^1^H COSY correlations starting from the anomeric proton, H-1/H-2 (*δ*_H_ 3.45, dd), H-2/H-3 (*δ*_H_ 3.67), H-3/H-4 (*δ*_H_ 3.38, t), and H-4/H-5 (*δ*_H_ 3.75). The corresponding carbon shifts were determined from HSQC as C-2 (71.12 ppm), C-3 (72.54 ppm), C-4 (69.15 ppm), C-5 (72.38 ppm), and C-6 (60.04 ppm).

The glycosidic linkage sequence of PGP-1 was analyzed using the HMBC spectrum (Fig. 3E). A key inter-residue cross-peak at *δ*_H_ 5.34/*δ*_C_ 103.18 (G H-1/F C-2) demonstrated that O-1 of the terminal α-D-Glc*p* was linked to C-2 of the adjacent →1)-β-D-Fru*f*-(2→ residue [36]. Furthermore, the cross-peak at *δ*_H_ 3.84/*δ*_C_ 103.18 (F H-1/F C-2) confirmed that the →1)-β-D-Fru*f*-(2→ residues are interconnected through β-(2→1) glycosidic bonds, forming the repeating backbone of the inulin-type fructan. This observation is fully consistent with the methylation results, in which 1,2-Fru*f* was identified as the overwhelmingly predominant linkage type. Cross-peaks observed in the *δ*_H_ 3.75–3.85 region with the C-2 signals were attributed to intraresidue H-5/C-2 long-range correlations within the fructofuranose ring. The minor ^13^C signals observed adjacent to the major resonances (103.03 and 103.64 ppm in the C-2 region) are attributed to the terminal β-D-Fru*f*-(2→ and the →1)-β-D-Fru*f*-(2→ residue adjacent to the terminal Glc*p*, which together constitute less than 20% of total fructose residues. Due to their low signal intensity and substantial spectral overlap with the dominant repeating unit, these minor resonances were not independently assigned, consistent with established practice for linear inulin-type fructans [37].

Based on the integrated evidence from monosaccharide composition, methylation analysis, FT-IR spectroscopy, and 1D/2D NMR spectroscopy, the primary structure of PGP-1 was established as a linear inulin-type fructan (Fig. 3G). This structure is consistent with previously reported fructans from *Platycodon grandiflorus* [17, 38]. However, the immunomodulatory and indirect tumoricidal mechanisms of this structurally defined homogeneous fructan have not been previously investigated and are explored in the following sections.

#### SEM analysis of PGP-1

As shown in Fig. 3H–J, the SEM images of PGP-1 reveal a flaky stacking structure. Upon magnification, the surface appeared generally smooth with slight wrinkles. The layers were tightly interconnected, with pores of varying sizes at the junctions. These results show that PGP-1 has a highly homogeneous macrostructure.

### PGP-1 induces M1-like macrophage polarization

#### PGP-1 induces robust immune responses in macrophages

**Fig. 4:**
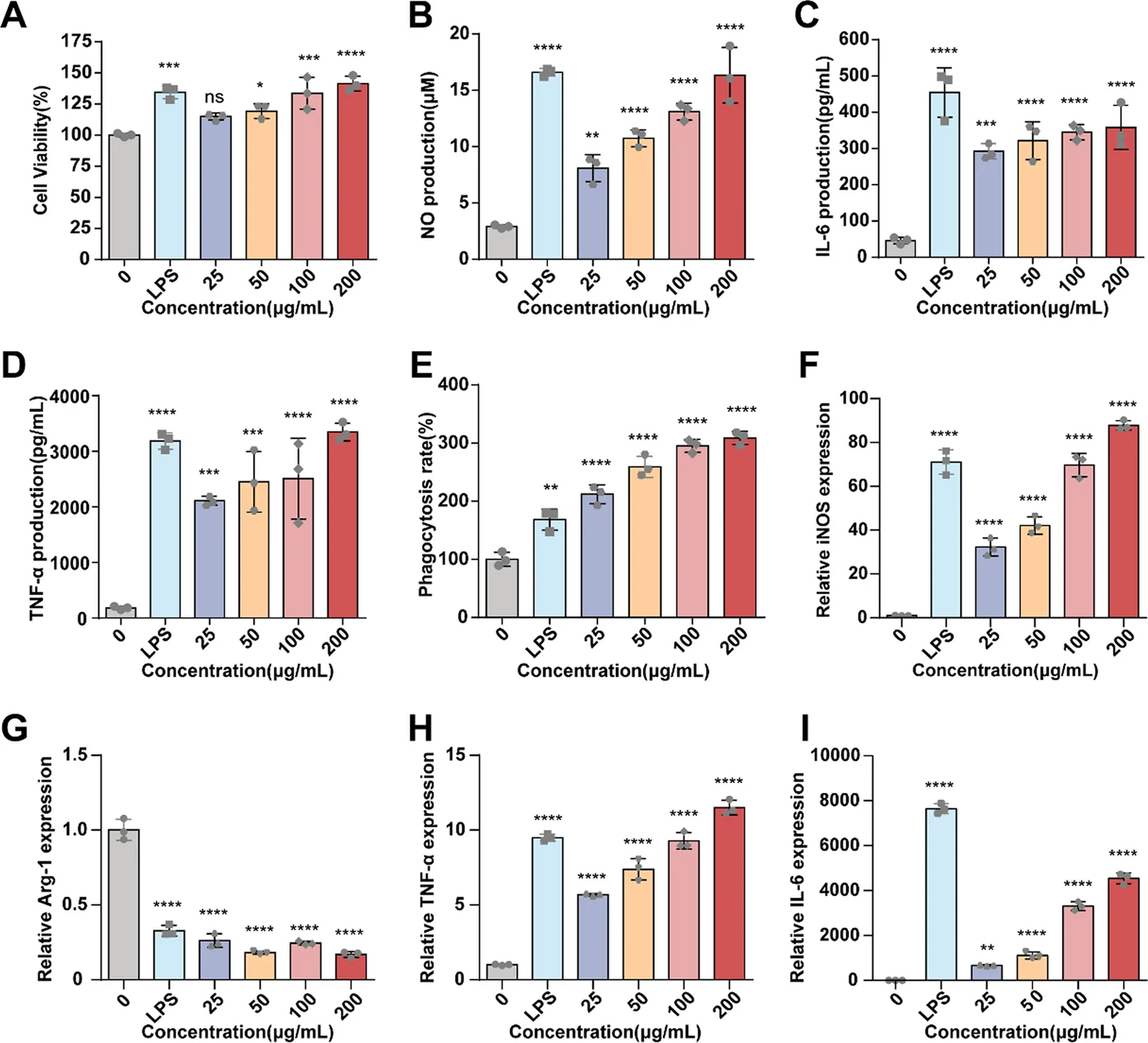
PGP-1 induces robust immune responses in macrophages. **A**–**E** PGP-1 induces immune responses in macrophages. Proliferation effect (**A**), NO (**B**), IL-6 (**C**), TNF-α (**D**) productions, and phagocytosis effect (**E**). Real-time PCR analysis of iNOS (**F**), Arg-1 (**G**) and inflammatory cytokines (TNF-α and IL-6) (**H**, **I**). 0 for control group. (n = 3 biological replicates, one-way ANOVA). Data are means ± SD. *P < 0.05, **P < 0.01, ***P < 0.001, ****P < 0.0001 *versus* control

Previous reports have shown that *Platycodon grandiflorus* polysaccharides possess immunomodulatory activity [12, 15]. Since PGP-1 was identified as the main purified component, we investigated its specific role in modulating macrophage behavior (Fig. S2). The effect of PGP-1 on RAW264.7 cell viability was examined by CCK8 assay and shown in Fig. 4A. Cell viability increased in a concentration-dependent manner after treatment of RAW264.7 cells with 25–200 μg/mL of PGP-1 for 48 h compared with the control. This indicated that PGP-1 markedly promotes the proliferation of RAW264.7 cells. As critical signaling molecules, cytokines are essential for tissue regeneration, cellular proliferation, and immune homeostasis in biological systems. Activated macrophages produce biologically active cytokines such as NO, TNF-α, IL-6 [39]. The secretion and release of NO enhances macrophage metabolic activity and phagocytic function, thereby potentiating immune responses [40]. Concurrently, TNF-α and IL-6 not only promote macrophage proliferation and tumor cell cytotoxicity but also serve as critical mediators of signal transduction and immune homeostasis maintenance [41]. As shown in Fig. 4B–D, PGP-1 significantly induced NO production and increased the levels of TNF-α and IL-6. An important indicator of immune evaluation is the phagocytic function of macrophages [26]. The effect of PGP-1 on phagocytosis in RAW264.7 cells was assessed using the neutral red uptake assay (Fig. 4E), the phagocytic capacity of RAW264.7 cells increased with increasing concentrations of PGP-1.

To rule out the contribution of endotoxin contamination to the observed immunostimulatory effects, the endotoxin content in purified PGP-1 was determined by gel-clot LAL assay. The results showed that PGP-1 at both 1 mg/mL and 200 μg/mL (the maximum working concentration) did not induce gel formation, indicating that the endotoxin level was below the detection limit of 0.125 EU/mL (Fig. S6A). A spike recovery test confirmed that PGP-1 did not interfere with the LAL reaction, validating the reliability of this result. Furthermore, polymyxin B (PMB) pretreatment completely abolished LPS-induced NO production (Fig. S6B), confirming the efficacy of PMB in neutralizing LPS. In contrast, PMB pretreatment did not significantly affect PGP-1-induced NO production (Fig. S6B), demonstrating that the immunomodulatory activity of PGP-1 is intrinsic to the polysaccharide molecule itself rather than arising from endotoxin contamination.

#### PGP-1 selectively promotes M1 macrophage polarization

**Fig. 5:**
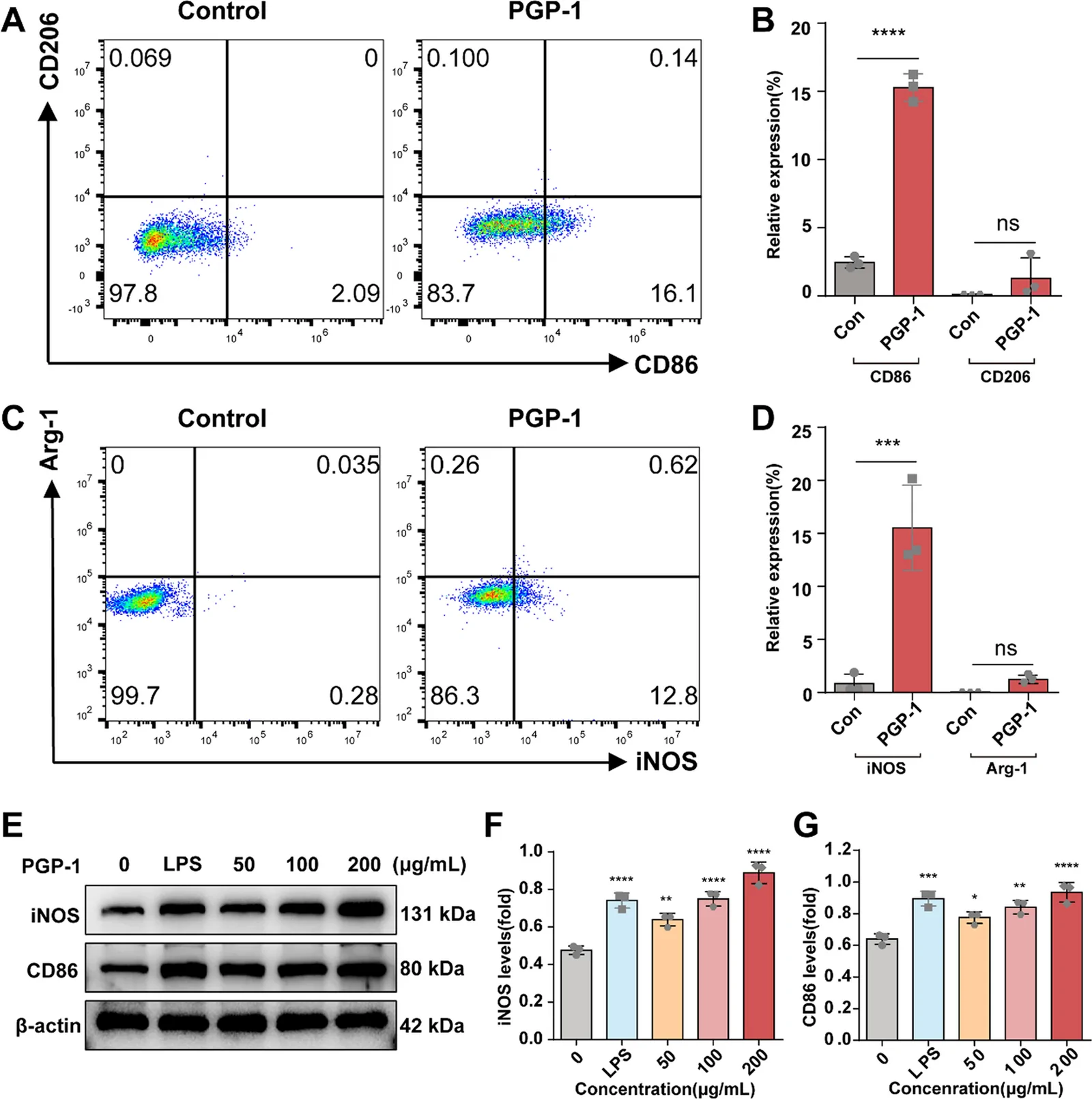
PGP-1 selectively promotes M1 macrophage polarization. **A**–**D** PGP-1 selectively promotes M1 macrophage polarization. Membrane biomarkers CD86 and CD206 (**A**, **B**), Membrane biomarkers iNOS and Arg-1 (**C**, **D**). **E**–**G** PGP-1 upregulated the expression of the M1-associated markers CD86 and iNOS proteins in RAW 264.7 cells. 0 for control group. (n = 3 biological replicates, one-way ANOVA). Data are means ± SD. *P < 0.05, **P < 0.01, ***P < 0.001, ****P < 0.0001 *versus* control

Macrophages are plastic cells that can be divided into M1 and M2 types in the tumor microenvironment [16]. M1 macrophages play an active role in the clearance of pathogens and tumor cells. In contrast, M2 macrophages suppress inflammation and promote tumor growth. There is evidence that M1 macrophages secrete tumor-killing molecules such as NO, TNF-α, and ROS, which have cytotoxic effects on tumor cells [42]. Cytokines such as IL-6, IL-12, and TNF-α, as well as surface proteins, CD86, CD80 and CD64, are well-established markers of M1 macrophages [43]. PGP-1 activated macrophages to secrete various pro-inflammatory cytokines and exerted immunomodulatory effects. To evaluate the inductive effect of PGP-1 on M1 polarization of RAW 264.7 cells, the expression of M1/M2‑related markers was determined at both mRNA and protein levels. Quantitative real‑time PCR analysis revealed that PGP-1 significantly upregulated the mRNA expression of M1‑related markers including iNOS, IL-6, and TNF‑α, whereas it exerted no significant effect on the M2 marker Arg‑1 (Fig. 4F–I). Consistent with the PCR results, flow cytometric analysis demonstrated that PGP-1 selectively enhanced the expression of M1 macrophage polarization markers (CD86 and iNOS) without significantly affecting the M2 phenotypic markers (CD206 and Arg‑1), as shown in Fig. 5A–D. Furthermore, Western blot analysis (Fig. 5E–G) revealed that PGP-1 treatment markedly increased the protein levels of CD86 and iNOS in RAW 264.7 macrophages compared with the control group, further confirming its ability to promote M1 phenotypic polarization.

### PGP-1 inhibits tumor cell activity indirectly by promoting macrophage activation

**Fig. 6:**
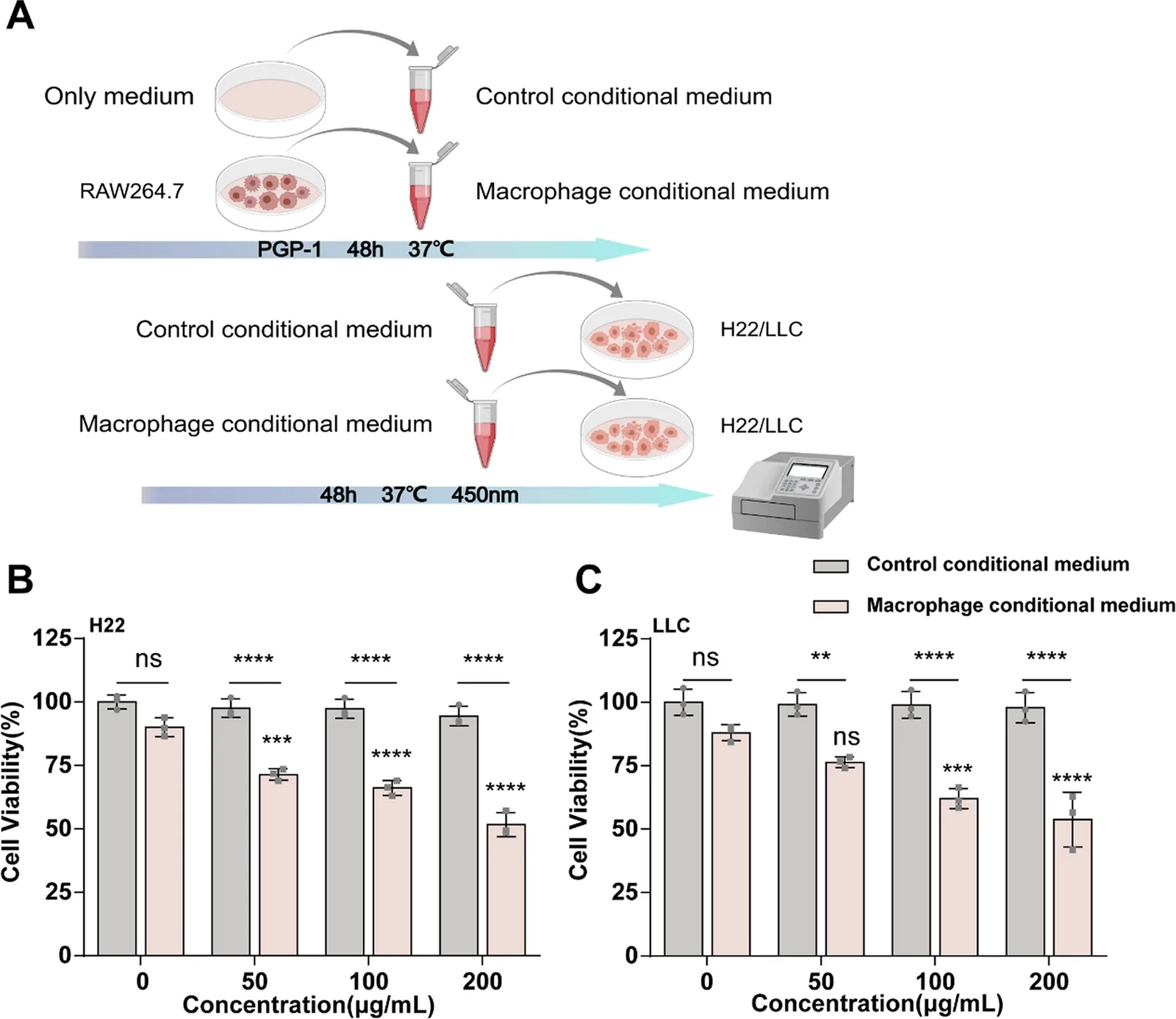
**A** Schematic diagram of conditional medium. **B**, **C** Cell viability of H22 (**B**) and LLC (**C**) cells after conditional medium stimulation. Negative control (macrophage conditional medium with 0 μg/mL PGP-1). (n = 3 biological replicates, one-way ANOVA). Data are means ± SD. *P < 0.05, **P < 0.01, ***P < 0.001, ****P < 0.0001 compared with the negative group

To determine whether PGP-1 exerts direct cytotoxicity against tumor cells or functions primarily as an immunomodulator, preliminary direct co-incubation assays were performed. At immunomodulatory concentrations (up to 200 μg/mL), PGP-1 alone exhibited negligible direct inhibitory effects on the viability of H22 and LLC cancer cells (48 h), indicating a lack of non-specific cellular toxicity Fig. S3. Therefore, we hypothesized that its tumoricidal efficacy is mediated through macrophage activation. Research has shown that inflammatory mediators such as NO and TNF-α, which are secreted by activated macrophages, directly promote the death of tumor cells [44]. As shown in Fig. 6A, two types of cancer cells, H22 and LLC, were treated with conditional medium containing cytokines produced by PGP-1-stimulated macrophages. As demonstrated in Fig. 6B, C, macrophage-conditioned medium containing PGP-1-derived factors suppressed viability of H22 hepatocellular carcinoma cells and LLC lung cancer cells by 51.71% and 53.77% at 200 μg/mL, respectively.

**Fig. 7:**
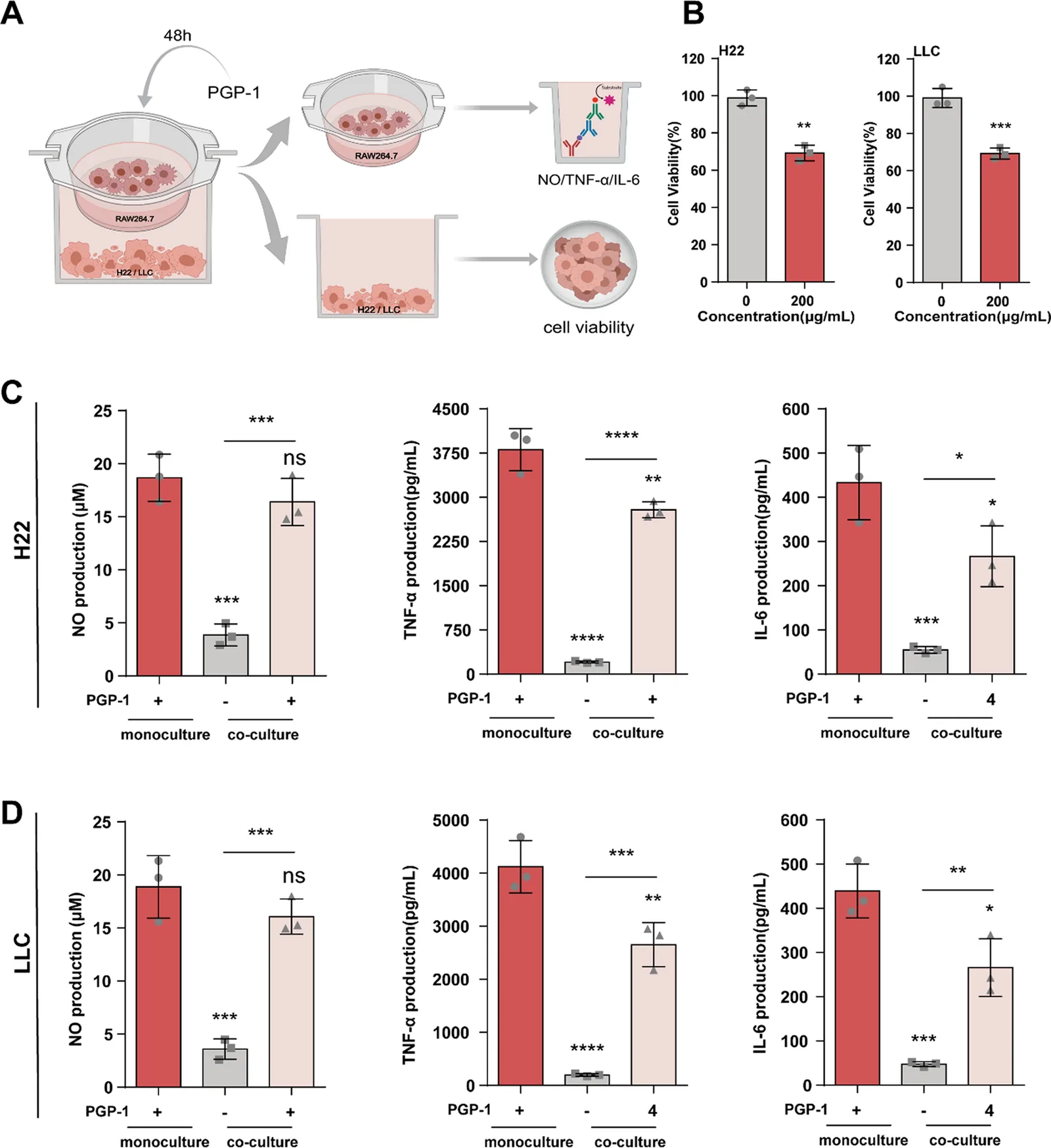
PGP-1 inhibits tumor cell activity indirectly by promoting macrophage activation. **A** co-culture system. RAW264.7 macrophages were co-cultured with H22 or LLC cell for 48 h in the presence or absence of PGP-1 (200 μg/mL). **B** Effect of co-culture on H22 or LLC cell viability. Negative control (macrophages treated with 0 μg/mL PGP-1 in co-culture system). **C**, **D** Evaluation of cytokines released by RAW264.7 macrophages treated with 200 μg/mL of PGP-1 in the co-culture system. Negative control (macrophages treated with 200 μg/mL PGP-1 in monoculture system). (n = 3 biological replicates, *t* test or one-way ANOVA). Data are means ± SD. *P < 0.05, **P < 0.01, ***P < 0.001, ****P < 0.0001 compared with the negative group

An in *vitro* model based on a transwell co-culture system that enables cells to communicate through small molecule channels was created to investigate the interaction between macrophages and cancer cells (Fig. 7A). The viability of tumor cells co-cultured with macrophages in the presence of PGP-1 (200 μg/mL) was reduced by approximately 30% compared to negative controls (Fig. 7B). On the other hand, in the co-culture system without PGP-1, it was clearly observed that the existence of tumor cells induced the macrophages towards the M2 state through low production of pro-inflammatory cytokines. As shown in Fig. 7C, D, levels of M1 markers (IL-6 and TNF-α) in the co-culture system were significantly lower than those in direct macrophage stimulation with the same PGP-1 dose (200 μg/mL), but still higher than in PGP-1-free co-cultures. These suggested that although tumor cells influenced macrophages towards an M2 phenotype, PGP-1 treatment established a persistent M1 state environment and contributed to suppress tumor cell viability. Collectively, these results indicated that PGP-1 inhibits tumor cell proliferation primarily through stimulating macrophage activation rather than exerting direct cellular toxicity.

### PGP-1 promotes macrophage activation through the TLR4-NF-κB pathway

**Fig. 8:**
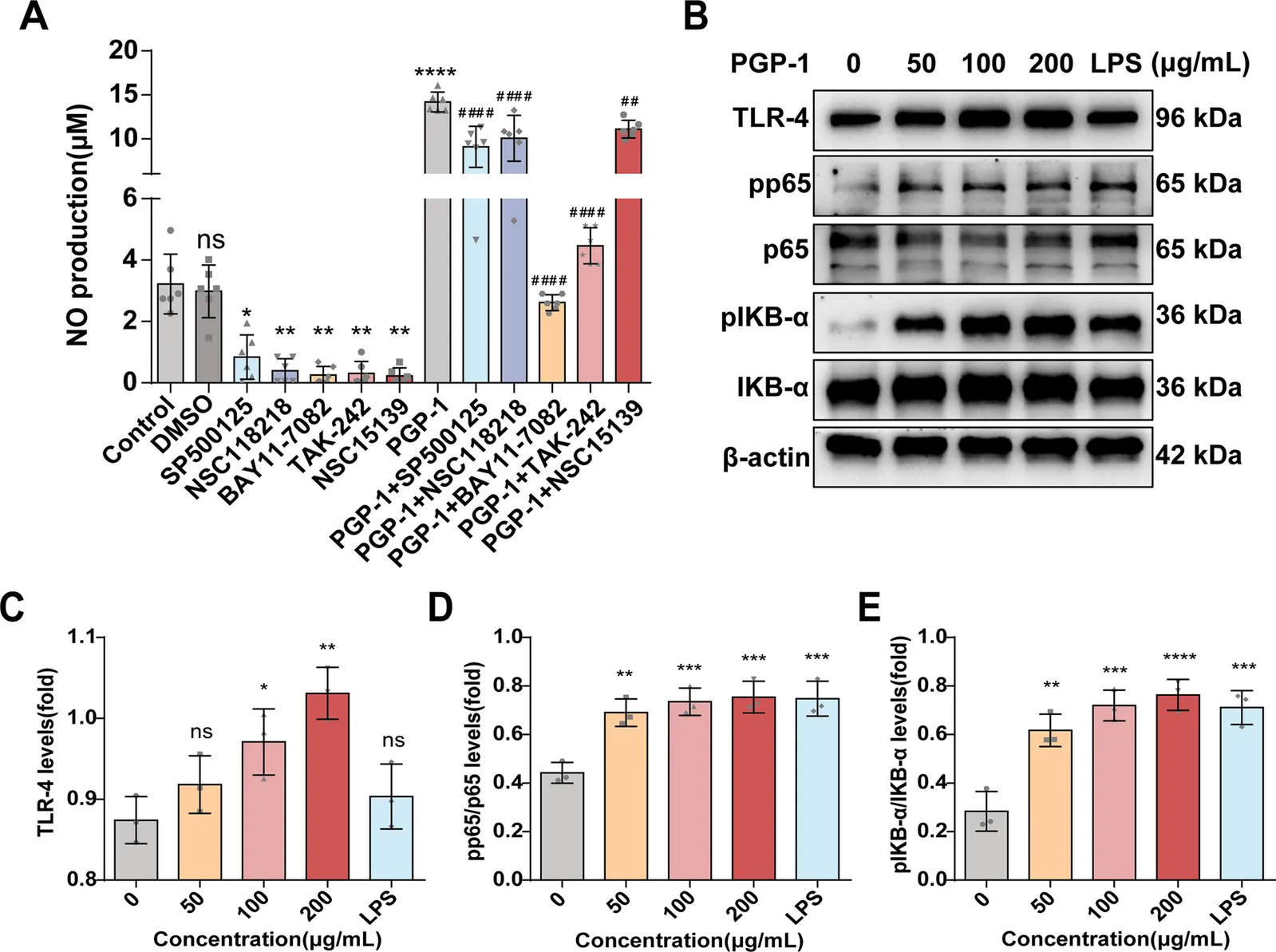
PGP-1 promotes macrophage activation through the TLR4-NF-κB pathway. **A** The inhibitory effects of specific signaling pathway antagonists on NO production were comparatively evaluated in both PBS-treated control macrophages and PGP-1-stimulated (200 μg/mL) cells. **B**–**E** PGP-1 treatment increased in the expression levels of TLR-4 (**C**), pp65/p65 (**D**) and pIKB-α/IKB-α (**E**) *versus* control. 0 for control group. *P < 0.05, **P < 0.01, ***P < 0.001, ****P < 0.0001 compared with the control group. #*P* < 0.05, ##*P* < 0.01, ###*P* < 0.001, ####*P* < 0.0001 *versus* PGP-1 group

As shown in Fig. 8A, all inhibitors tested reduced NO secretion compared with the control group. In addition, NO production was increased in macrophages stimulated with PGP-1 after pretreatment with a specific inhibitor relative to the inhibitor-only group, but the levels were still significantly lower than those of PGP-1 treatment alone. Notably, among the tested inhibitors, BAY11-7082 (NF-κB inhibitor) and TAK-242 (TLR-4 inhibitor) demonstrated the most potent suppression, reducing NO secretion to near-baseline levels comparable to untreated controls. These results showed that PGP-1-promoted macrophage activation was inhibited by TLR-4 and NF-κB pathway inhibitors. In order to determine that PGP-1 promotes macrophage activation through the TLR4-NF-κB pathway, the expression levels of TLR-4, pp65/p65, and pIKB-α/IKB-α in PGP-1-treated RAW264.7 cells were detected by Western blotting. As shown in Fig. 8B–E, PGP-1 treatment resulted in a significant increase in the expression levels of TLR-4, pp65/p65 and pIKB-α/IKB-α compared with untreated cells. The above results suggested that PGP-1 promoted macrophage activation through the TLR4-NF-κB pathway.

## Discussion

Herein, we characterized the structural properties of PGP-1, a bioactive polysaccharide isolated from *Platycodon grandiflorus*, and investigated its immunomodulatory tumoricidal mechanisms mediated by macrophage activation. PGP-1 was composed of fructose and glucose, with the predominant glycosidic linkage identified as →1)-β-D-Fru*f*-(2→, together with terminal β-D-Fru*f*-(2→ and terminal α-D-Glc*p*-(1→, characteristic of an inulin-type fructan. This structural assignment is consistent with previously reported *Platycodon grandiflorus* fructans from Anhui Province, which are known for the predominant β-(2→1) linkage [17, 38].

It should be noted that a trace amount of →1,6)-β-D-Fru*f*-(2→ (2.19%) was detected in the methylation analysis. However, NMR spectra did not reveal diagnostic signals for significant 6-O-glycosylation at the C-6 position of fructose residues. During the course of this study, an initial methylation experiment yielded a higher proportion of →1,6)-β-D-Fru*f*-(2→ (9%), but a repeat experiment under identical conditions reduced this value to 2.19%, which was more consistent with the NMR data. The discrepancy between the two methylation experiments may be attributed to under-methylation in the initial experiment, a recognized technical limitation in fructan methylation analysis where incomplete methylation of free hydroxyl groups can generate false-positive signals for multi-substituted linkages [29]. The detailed data from both methylation experiments are provided in the Supplementary Materials (Table S5 and Fig. S5).

Due to the structural lability of fructose residues, PGP-1 was hydrolyzed under mild conditions with 3 M TFA at 60 °C for 3 h for monosaccharide composition analysis. This method allowed fructans to be largely retained as fructose residues, although partial degradation may still occur, accounting for the slight discrepancies between the monosaccharide composition (Glc:Fru = 1:9.45), methylation peak area ratios, and the molecular weight. Such discrepancies are inherent to the semi-quantitative nature of methylation analysis and the susceptibility of fructose to acid hydrolysis [35, 45]. Despite these methodological limitations, all three analytical approaches consistently point to the same fundamental structure: a low-molecular-weight inulin-type fructan predominantly composed of fructose with a single terminal glucose.

A major hurdle in contemporary oncology is the severe collateral damage and dose-limiting toxicity associated with conventional chemotherapeutics, which predominantly rely on non-specific cytotoxicity [46]. To overcome these limitations, targeting TME, specifically the highly plastic tumor-associated macrophages (TAMs), has emerged as a cutting-edge therapeutic strategy [16]. In a typical TME, cancer cells often hijack TAMs, skewing them toward an immunosuppressive, pro-tumorigenic M2-like phenotype that facilitates tumor immune evasion, angiogenesis, and metastasis [47]. Therefore, reprogramming TAMs from the M2 state back toward the tumoricidal M1 phenotype is crucial for restoring anti-tumor immunity. Unlike traditional cytotoxic agents, naturally derived macromolecules, particularly plant polysaccharides, offer a fundamentally different and safer therapeutic paradigm by serving as targeted immunomodulators that dictate this M1 polarization [48, 49]. This paradigm effectively circumvents the toxic side effects associated with conventional therapies, achieving an optimal balance between therapeutic efficacy and safety.

**Fig. 9:**
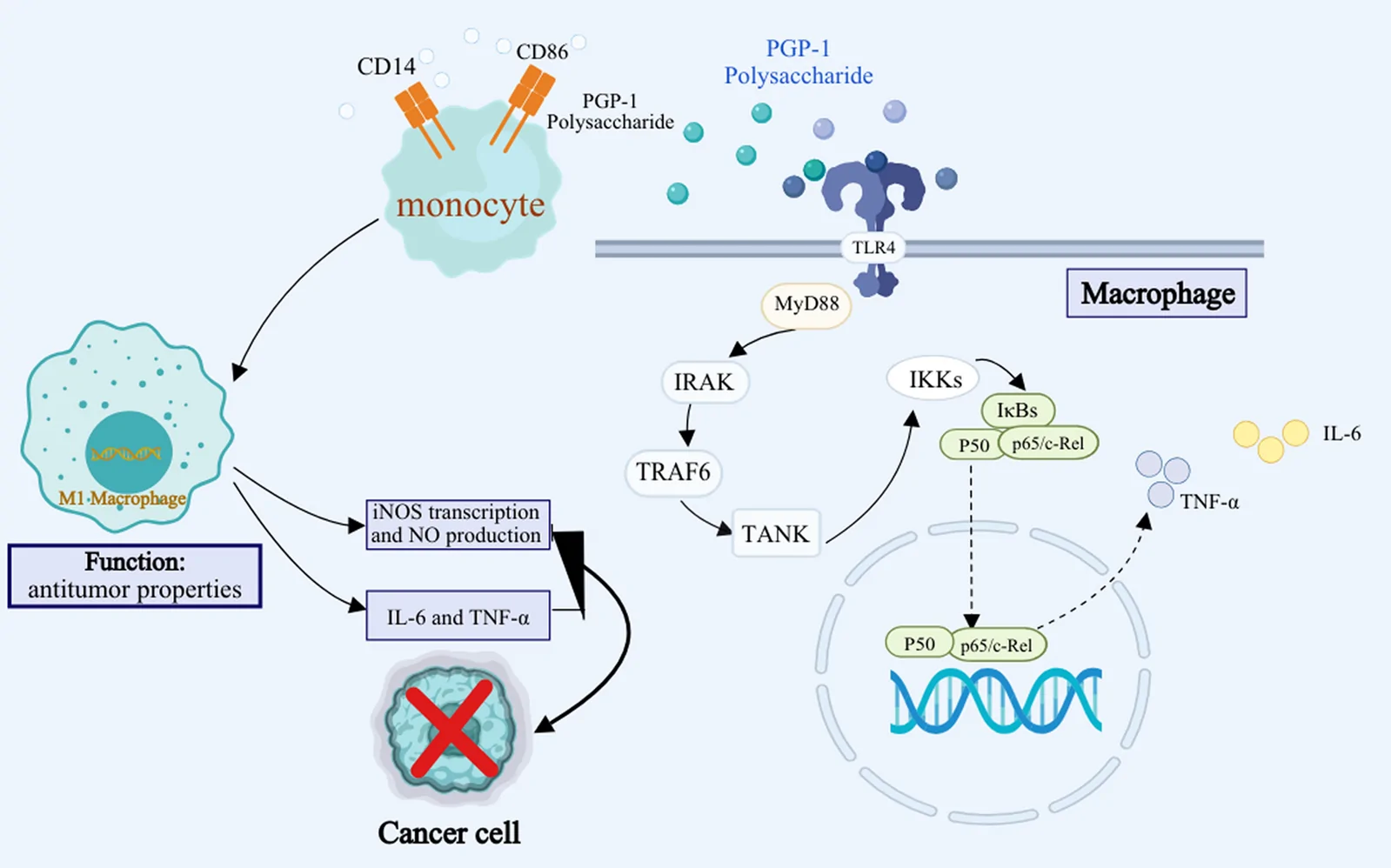
The potential mechanisms of PGP-1 inhibited cancer cell proliferation

The findings from our pharmacological evaluations directly address the scientific inquiry raised in the introduction regarding the mechanistic integration of the polysaccharide's safety profile with its potent tumoricidal efficacy. Pharmacological evaluations demonstrated that the tumoricidal efficacy of PGP-1 is primarily mediated through remodeling the immune microenvironment rather than exerting direct cellular toxicity (Fig. 9). In the absence of macrophages, PGP-1 exhibited negligible direct toxicity against H22 and LLC cancer cells even at high functional doses. This experimental observation is highly consistent with its inherent safety characteristics as a botanical derivative from a medicinal and edible plant. However, the transwell co-culture model, which effectively simulates the complex intercellular cross-talk within the TME, revealed a dramatic shift. Once introduced into the co-culture system, PGP-1 effectively counteracted the tumor-induced immune suppression, restoring and sustaining a robust M1 macrophage phenotype. These activated macrophages subsequently released a barrage of tumoricidal factors, prominently NO, TNF-α, and IL-6, which are well-documented to induce cancer cell apoptosis and inhibit proliferation [44]. This targeted "indirect killing" mechanism maximizes localized anti-tumor immune responses while entirely bypassing the risk of damaging normal somatic cells, thereby providing a compelling mechanistic explanation for how PGP-1 exerts remarkable tumor-suppressive efficacy while maintaining minimal cytotoxicity.

Furthermore, we demonstrated that this macrophage repolarization is driven by the classical TLR4/NF-κB signaling axis. TLR4 functions as a critical pattern recognition receptor (PRR) on the macrophage surface. Recent evidence indicates that specific structural features of plant polysaccharides allow them to act as pathogen-associated molecular patterns (PAMPs), directly binding to TLR4 and triggering downstream MyD88-dependent NF-κB activation [50]. Previous studies have established that inulin-type fructans rich in β-(2→1)-linked fructose residues possess potent macrophage-stimulating activities and can be directly recognized by TLR4 receptors [51, 52]. The activation of the TLR4/NF-κB axis by PGP-1 is consistent with these findings and suggests that the β-(2→1)-linked fructose backbone of PGP-1 may serve as the structural basis for TLR4 recognition. These findings highlight the potential of PGP-1 as a structurally characterized, low-toxicity natural immune enhancer for cancer immunotherapy.

This study characterized the structure of a *Platycodon grandiflorus* polysaccharide (PGP-1) as an inulin-type fructan and provided preliminary evidence for its immune-based antitumor efficacy. Through the integration of experimental evidence and theoretical analysis, we achieved a mechanistic link between PGP-1's safety profile and its significant tumoricidal activity. Further research is warranted to investigate its synergistic effects with other immune cells in cancer treatment in vivo. Additionally, the precise molecular docking and binding kinetics between the inulin-type fructan structure and the TLR4 receptor require further investigation. In summary, these findings suggest that PGP-1 has potential for development as a low-toxicity natural immunomodulator.

## Conclusion

This research investigated the structural characteristics and immunomodulatory mechanisms of PGP-1, a homogeneous polysaccharide derived from *Platycodon grandiflorus*. Structural characterization revealed an inulin-type fructan predominantly composed of →1)-β-D-Fru*f*-(2→ as the main chain, with terminal β-D-Fru*f*-(2→ and α-D-Glc*p*-(1→ residues. Biological evaluations demonstrated that PGP-1 promoted macrophage proliferation, stimulated macrophages to produce key inflammatory cytokines (NO, IL-6, TNF-α), and enhanced their phagocytic activity. Further mechanistic studies confirmed that PGP-1 drives macrophage M1 polarization via the TLR4-NF-κB signaling pathway. Importantly, endotoxin contamination was excluded as a contributing factor through LAL assay and polymyxin B neutralization experiments. Crucially, this study established a mechanistic link between the polysaccharide's safety profile and its tumor-suppressive efficacy: experimental evidence confirmed that PGP-1 exhibits negligible direct cytotoxicity at 200 μg/mL, yet effectively inhibits cancer cell proliferation indirectly by augmenting macrophage-mediated tumoricidal activity in a co-culture model. In conclusion, this investigation provides preliminary evidence for the macrophage-targeted mechanism of PGP-1 in inhibiting tumor growth. These findings contribute to the bioprospecting of *Platycodon grandiflorus* polysaccharides and provide astructural and mechanistic basis for the development of PGP-1 as a high-efficiency and low-toxicity natural immune enhancer.

## Supplementary Information

Supplementary Material 1.

## Data Availability

Data will be made available on request.
