# Supplementary material for: Isolation, structural characterization, immunomodulatory and indirect tumoricidal activities of an inulin-type fructan from Platycodon grandiflorus via macrophage M1 polarization through the TLR4/NF-κB pathway
Source: Nat Prod Bioprospect. 2026 Nov 2;16(1):99. doi: 10.1007/s13659-026-00657-5 (PMC13728019; doi:10.1007/s13659-026-00657-5)
Supplement: Supplementary file 1 [file 13659_2026_657_MOESM1_ESM.docx]

Supplementary data for

**Isolation, structural characterization, immunomodulatory and indirect tumoricidal activities of an inulin-type fructan from *Platycodon grandiflorus* via macrophage M1 polarization through the TLR4/NF-κB pathway**

Chen-Xi Wang^a,d,1^, Ping-Yan Liao^b,c,1^, Cui-Lian Yin^a^, Yue Wang^a^, Jie Hu^a^, Kui Li^a^, Zhao-Kun Li^a^, Yu-Jie Sun^a^, Xian-Yang Li^a^, Jiang-Miao Hu^d,*^, Ning Li^a,*^, Meng-Ting Kuang^a,*^

a Anhui Key Laboratory of Bioactivity of Natural Products, School of Pharmacy, Anhui Medical University, Hefei 230032, China.

b The Tenth Clinical Medical College of Guangzhou University of Traditional Chinese Medicine, Zhongshan, 528400, China.

c Zhongshan Hospital of Traditional Chinese Medicine, Zhongshan, 528400, China.

d State Key Laboratory of Phytochemistry and Natural Medicines, Kunming Institute of Botany, Chinese Academy of Sciences, Kunming 650201, China.

*Corresponding author: kuangmengting@ahmu.edu.cn; 1993500019@ahmu.edu.cn; hujiangmiao@mail.kib.ac.cn;

1 These authors contribute equally to this work

**Catalogue**

[Supplementary methods	4](#_Toc234954113)

[Tables	8](#_Toc234954114)

[Table S1. Range and corresponding levels of independent variables.	8](#_Toc234954115)

[Table S2. Box‒Behnken design (BBD) matrix of the four variables.	9](#_Toc234954116)

[Table S3. Variance analysis of the fitted regression model equation.	10](#_Toc234954117)

[Table S4. Primers for qRT-PCR (mouse).	11](#_Toc234954118)

[Table S5. Methylation analysis of PGP-1.	12](#_Toc234954119)

[Figures	13](#_Toc234954120)

[Fig. S1. Effects of the four variables on the extraction rate of PGPs (%).	13](#_Toc234954121)

[Fig. S2. The HPGPC profile of crude PGPs.	14](#_Toc234954122)

[Fig. S3. PGP-1 alone exhibited negligible direct inhibitory effects on the viability of H22 and LLC cancer cells (48 h).	15](#_Toc234954123)

[Fig. S4. The monosaccharide composition analysis of PGP-1.	16](#_Toc234954125)

[Fig. S5. Methylation analysis of PGP-1.	17](#_Toc234954126)

[Fig. S6. Exclusion of endotoxin contamination.	18](#_Toc234954127)

[Fig. S7. NMR spectra of PGP-1.	20](#_Toc234954128)

[References	21](#_Toc234954129)

# Supplementary methods

**Single-factor test**

The single-factor test was performed first. The dried material was crushed and sieved through a 60 mesh sieve, soaked in acetone and ether at room temperature then baked at 45 ℃. The pretreated powder of *Platycodon grandiflorus* was mixed with pure water, the liquid-material ratio was adjusted to 10-30 mL/g, the temperature was adjusted to 40-120 ℃, and the extract was placed in a water bath for 30-150 min, then filtered through gauze. The filtrate was then extracted 1-5 times. The filtrates were combined and centrifugated at 4500 rpm for 10 min, then the supernatant was collected and concentrated to the appropriate concentration using a rotary evaporator. The concentrated supernatant was stirred with 95% ethanol to adjust the final alcohol concentration to 75%, sealed with plastic wrap and placed in a refrigerator at 4 ℃ overnight. Centrifugation was performed at 4500 rpm/min for 10 min to obtain the precipitate. The precipitate was dissolved in distilled water and lyophilized to obtain crude PGPs.

Based on the yield results (Fig. S1), the corresponding surface analyses (Table S1) were performed using factor A time (60, 90, and 120 min), factor B temperature (60, 80, and 100°C), factor C liquid-to-material ratio (10, 15, and 20 mL/g), and factor D number of extractions (1, 2, and 3 times). A Box-Behnken design with 29 runs was used for statistical optimization of PGPs extraction (Table S2).

**Effect of heating time, temperature, liquid/material ratio and extraction times on PGPs extraction**

The extraction rate of PGPs exhibited a gradual increase from the initial heating time of 30 min to 90 min, reaching a maximum at the latter time point (Fig. S1A). Extraction times exceeding 120 min led to an increase in polysaccharide extraction, but this increase was not significant and resulted in a concomitant increase in impurity extraction. Conversely, extraction times below 60 min resulted in incomplete extraction.

The extraction rate of PGPs exhibited a nearly one-fold increase with elevated heating temperatures (40 °C-80 °C), reaching a maximum at 80 °C (Fig. S1B). In general, an increase in temperature results in an elevated kinetic energy of solvent molecules, thereby facilitating enhanced interaction between the sample and the solvent. Nevertheless, the continuously increasing temperature degrades thermophilic compounds. Besides, the solubility and diffusivity of the solvent decrease rapidly with temperature reaching the boiling point of the solvent. The above reasons may cause a significant reduction in the extraction rate. ^[1]^.

The extraction rate of PGPs varied insignificantly with an increasing liquid-material ratio (Fig. S1C). From the results, it can be obtained that increasing the liquid/material ratio on the basis of 10 mL/g will not significantly increase the polysaccharide extraction rate, but cause inconvenience in concentration.

The PGPs extraction rate showed an increasing and then decreasing trend as the number of extractions increased, the highest extraction rate was performed with twice extractions (Fig. S1D). The reason may be that once extraction was not enough, while too many extraction times lead to an increase in impurity amount.

In conclusion, the optimal ranges of time (min), temperature (°C), liquid/material ratio (mL/g) and times (n) for PGPs extraction were found to be 60-120 min, 60-100 °C, 10-20 mL/g and 1-3 times, respectively.

**Fitting the model**

Response surface analysis is used to optimize the extraction conditions of target compounds of natural products, it is one of the most popular multivariate statistical techniques ^[2]^. Therefore, this method was used in the present study to optimize the PGPs extraction process with four variables (time, temperature, liquid-feed ratio and number of extractions) mentioned above. A polynomial equation for predicting PGPs extraction rate (Y) was listed below using Box-Behnken.

Y = 18.07 + 1.32A - 1.47B + 0.35C + 0.72D - 0.50AB + 0.095AC + 0.58AD + 1.76BC + 0.11BD - 0.83CD - 1.86 A^2^ - 3.81 B^2^ - 1.01 C^2^ - 4.69 D^2^

In this equation, A, B, C and D presented heating time, heating temperature, liquid/material ratio and number of extractions, respectively.

As shown in Table S3, the low *P value* (< 0.01) and high *F value* (> 10) indicated that the quadratic polynomial model for PGPs extraction rate was statistically significant ^[3]^. R^2^_adj_, an index used to measure the correlation extent between predicted values and observed values, was 0.9164 in this fitted regression model equation, which indicated that 91.64% of the variation could use this model to explain ^[4]^. The *F value* and *P value* of the misfit model were 0.74 and 0.6803 (> 0.05), respectively, and the efficiency of variation (CV) value was 6.94, indicating good accuracy and reliability of the experiment. In conclusion, according to the ANOVA results, the model accurately described data variation.

The twice terms (A^2^, B^2^, C^2^, D^2^) of four independent variables, the linear terms (A, B, D) for the three independent variables of time, temperature and number of extractions, and the interaction terms for temperature (B) and liquid-material ratio (C) were significant (*p* < 0.05) (Table S3). The smaller *P values* and greater *F values* mean the more significant variables ^[3, 5]^. Thus, the variable that had the greatest effect on PGPs extraction rate was heating temperature (*F* at 30.28, *p* < 0.0001), followed by heating time (*F* at 24.48, *p* at 0.0002) and number of extractions (F of 7.28, *p* at 0.0173), and lastly, liquid-material ratio (*F* at 1.68, *p* at 0.2159). The above results showed that the main factor affecting the yield of PGPs was the heating temperature (B), and the independent variables in decreasing order affect the extraction rate: temperature > time > number of extractions > liquid/ material ratio.

# Tables

## Table S1. Range and corresponding levels of independent variables.

| Coded factor level | Heating time (min) (A) | Heating temperature (℃) (B) | Liquid to material ratio (mL/g) (C) | Number of extractions (n) (D) |
| --- | --- | --- | --- | --- |
| -1 | 60 | 60 | 10 | 1 |
| 0 | 90 | 80 | 15 | 2 |
| 1 | 120 | 100 | 20 | 3 |

## Table S2. Box‒Behnken design (BBD) matrix of the four variables in coded units and response values for the extraction yield of PGPs.

| Number | (A) Heating time (min) | (B) Heating temperature (℃) | (C) Liquid to material ratio (mL/g) | (D)Number of extractions (n) | Extraction  rate (%) |
| --- | --- | --- | --- | --- | --- |
| 1 | 60 | 60 | 15 | 2 | 12.51 |
| 2 | 120 | 60 | 15 | 2 | 15.99 |
| 3 | 60 | 100 | 15 | 2 | 9.52 |
| 4 | 120 | 100 | 15 | 2 | 11.02 |
| 5 | 90 | 80 | 10 | 1 | 10.09 |
| 6 | 90 | 80 | 20 | 1 | 13.11 |
| 7 | 90 | 80 | 10 | 3 | 13.01 |
| 8 | 90 | 80 | 20 | 3 | 14.71 |
| 9 | 60 | 80 | 15 | 1 | 9.32 |
| 10 | 120 | 80 | 15 | 1 | 12.01 |
| 11 | 60 | 80 | 15 | 3 | 9.51 |
| 12 | 120 | 80 | 15 | 3 | 14.50 |
| 13 | 90 | 60 | 10 | 2 | 16.13 |
| 14 | 90 | 100 | 10 | 2 | 10.51 |
| 15 | 90 | 60 | 20 | 2 | 12.09 |
| 16 | 90 | 100 | 20 | 2 | 13.53 |
| 17 | 60 | 80 | 10 | 2 | 14.17 |
| 18 | 120 | 80 | 10 | 2 | 15.59 |
| 19 | 60 | 80 | 20 | 2 | 15.21 |
| 20 | 120 | 80 | 20 | 2 | 17.01 |
| 21 | 90 | 60 | 15 | 1 | 10.50 |
| 22 | 90 | 100 | 15 | 1 | 7.52 |
| 23 | 90 | 60 | 15 | 3 | 12.01 |
| 24 | 90 | 100 | 15 | 3 | 9.47 |
| 25 | 90 | 80 | 15 | 2 | 19.32 |
| 26 | 90 | 80 | 15 | 2 | 18.49 |
| 27 | 90 | 80 | 15 | 2 | 17.84 |
| 28 | 90 | 80 | 15 | 2 | 16.52 |
| 29 | 90 | 80 | 15 | 2 | 18.16 |

## Table S3. Variance analysis of the fitted regression model equation.

| Source of variance | | Sum of squares | Degree of freedom | | Mean square | | *F value* | *P value* | | Significance |
| --- | --- | --- | --- | --- | --- | --- | --- | --- | --- | --- |
| Model | | 275.39 | 14 | | 19.67 | | 22.92 | < 0.0001 | | *** |
| A-heating time | | 21.01 | 1 | | 21.01 | | 24.48 | 0.0002 | | *** |
| B-heating temperature | | 25.99 | 1 | | 25.99 | | 30.28 | < 0.0001 | | *** |
| C-liquid to material ratio | | 1.44 | 1 | | 1.44 | | 1.68 | 0.2159 | |  |
| D-number of extractions | | 6.25 | 1 | | 6.25 | | 7.28 | 0.0173 | | * |
| AB | | 0.98 | 1 | | 0.98 | | 1.14 | 0.3034 | |  |
| AC | | 0.036 | 1 | | 0.036 | | 0.042 | 0.8405 | |  |
| AD | | 1.32 | 1 | | 1.32 | | 1.54 | 0.2349 | |  |
| BC | | 12.46 | 1 | | 12.46 | | 14.52 | 0.0019 | | ** |
| BD | | 0.048 | 1 | | 0.048 | | 0.056 | 0.8157 | |  |
| CD | | 2.76 | 1 | | 2.76 | | 3.21 | 0.0948 | |  |
| A^2^ | | 22.55 | 1 | | 22.55 | | 26.27 | 0.0002 | | *** |
| B^2^ | | 94.14 | 1 | | 94.14 | | 109.67 | < 0.0001 | | *** |
| C^2^ | | 6.68 | 1 | | 6.68 | | 7.78 | 0.0145 | | * |
| D^2^ | | 142.66 | 1 | | 142.66 | | 166.19 | < 0.0001 | | *** |
| Residual | | 12.02 | 14 | | 0.86 | |  |  | |  |
| Lack of Fit | | 7.82 | 10 | | 0.78 | | 0.74 | 0.6803 | | Not significant |
| Pure Error | | 4.20 | 4 | | 1.05 | |  |  | |  |
| Cor Total | | 287.41 | 28 | |  | |  |  | |  |
|  | R^2^ = 0.9582 | | | R^2^_adj_ = 0.9164 | | R^2^_pred_ = 0.8205 | | | C.V. % = 6.94 | |

**p* < 0.05 considering as significant, ***p* < 0.01 considering as highly significant, and ****p* < 0.001 considering as extremely significant.

## Table S4. Primers for qRT-PCR (mouse).

| Terms | Forward primer (5'-3') | Reverse primer (5'-3')) |
| --- | --- | --- |
| IL-6 | CTTCTTGGGACTGATGCTGGTGAC | TCTGTTGGGAGTGGTATCCTCTGTG |
| TNF-α | CGCTCTTCTGTCTACTGAACTTCGG | GTGGTTTGTGAGTGTGAGGGTCTG |
| iNOS | GTTCTCAGCCCAACAATACAAGA | GTGGACGGGTCGATGTCAC |
| Arg-1 | CTCCAAGCCAAAGTCCTTAGAG | GGAGCTGTCATTAGGGACATCA |
| β-Actin | GGCTGTATTCCCCTCCATCG | CCAGTTGGTAACAATGCCATGT |

## Table S5. Methylation analysis of PGP-1.

| Linkages  types | Partially methylated  alditol acetates | Mass fragments (m/z) | Relative molar ratios (%) |
| --- | --- | --- | --- |
| t-Fru*f* | 1,3,4,6-tetra-O-methyl hexitols (mannitol, glucitol) | 87,101,102,129,161,162,205 | 10.92 |
| 1,2-Fru*f* | 3,4,6-tri-O-methyl hexitols (mannitol, glucitol) | 87,101,129,145,161,190 | 81.97 |
| 1,2,6-Fru*f* | 3,4-di-O-methyl hexitols (mannitol, glucitol) | 87,99,100,129,189,190 | 2.19 |
| t-Glc*p* | 2,3,4,6-tetra-O-methyl glucitol | 87,102,118,129,145,161,162,205 | 4.93 |

# Figures

## Fig. S1. Effects of different heating time (A), heating temperature (B), liquid to material ratio (C) and number of extractions (D) on the extraction rate of PGPs (%).

## Fig. S2. The HPGPC profile of crude PGPs.

## Fig. S3. PGP-1 alone exhibited negligible direct inhibitory effects on the viability of H22 and LLC cancer cells (48 h). (A-B) CCK8 assay. H22 (A), LLC (B). Cells were treated with PGP-1 for 48 h. 0 for control group. (n = 3 biological replicates, one-way ANOVA). Data are means ± SD. *P < 0.05, **P < 0.01, ***P < 0.001, ****P < 0.0001 versus control.

## Fig. S4. The monosaccharide composition analysis of PGP-1. The HPAEC-PAD chromatogram, standard monosaccharides (A), and PGP-1 (B). Standard curves for glucose (C) and fructose (D).

## Fig. S5. Methylation analysis of PGP-1. (A) Total ion chromatogram (TIC) of PMAAs derived from PGP-1. (B–E) EI-MS fragmentation spectra of each identified PMAA: (B) 1,3,4,6-tetra-O-methyl hexitols (terminal-Fruf); (C) 2,3,4,6-tetra-O-methyl glucitol (terminal-Glcp); (D) 3,4,6-tri-O-methyl hexitols (→1)-Fruf-(2→); (E) 3,4-di-O-methyl hexitols (→1,6)-Fruf-(2→). (F) Total ion chromatogram from the initial methylation experiment (see Discussion, Section 4).

Fig. S6. Exclusion of endotoxin contamination. (A) Gel-clot LAL assay results for PGP-1. (a) Negative control (endotoxin-free water): no gel formation (−); (b) Positive control (0.25 EU/mL LPS): gel formation (+); (c) PGP-1 (1 mg/mL): no gel formation (−); (d) Spike recovery: PGP-1 (1 mg/mL) + 0.25 EU/mL LPS: gel formation (+); (e) PGP-1 (200 μg/mL): no gel formation (−); (f) Spike recovery: PGP-1 (200 μg/mL) + 0.25 EU/mL LPS: gel formation (+). (B) Effect of polymyxin B (PMB, 30 μg/mL) pretreatment on NO production in RAW264.7 cells stimulated with LPS (1 μg/mL) or PGP-1 for 48 h. Data are means ± SD (n = 3 biological replicates, one-way ANOVA). **P* < 0.05, ***P* < 0.01, ****P* < 0.001, *****P* < 0.0001 *versus* control.

## Fig. S7. NMR spectra of PGP-1. 1H NMR spectrum (A), ^13^C NMR spectrum (B), DEPT-135 spectrum (C), ^1^H–^1^H COSY spectrum (D), HSQC spectrum (E), HMBC spectrum (F), ROESY spectrum (G) and TOCSY spectrum (H).

# References

[1]	S.U. Kadam, B.K. Tiwari, T.J. Smyth, et al., Optimization of ultrasound assisted extraction of bioactive components from brown seaweed *Ascophyllum nodosum* using response surface methodology, Ultrason Sonochem. 23 (2015) 308-316. https://doi.org/10.1016/j.ultsonch.2014.10.007.

[2]	A.E. Maccarronello, N. Cardullo, A.M. Silva, et al., From waste to bioactive compounds: A response surface methodology approach to extract antioxidants from *Pistacia* *vera* shells for postprandial hyperglycaemia management, Food Chem. 443 (2024) 138504. https://doi.org/10.1016/j.foodchem.2024.138504.

[3]	C.-Y. Gan, N.H.A. Manaf, A.A. Latiff, Optimization of alcohol insoluble polysaccharides (AIPS) extraction from the *Parkia speciosa* pod using response surface methodology (RSM), Carbohydr Polym. 79 (2010) 825-831. https://doi.org/10.1016/j.carbpol.2009.10.006.

[4]	Y. Liu, C. Guo, C. Wang, et al., Changes in the physicochemical properties and structural characteristics of rice bran polysaccharides extracted by specific enzyme cocktail and ultrasound, Food Chem. 476 (2025) 143453. https://doi.org/10.1016/j.foodchem.2025.143453.

[5]	C. Zhang, W. Ding, A. Mamattursun, et al., Optimization of enzyme-ultrasound assisted extraction from mulberries anthocyanins based on response surface methodology and deep neural networks and analysis of in vitro antioxidant activities, Food Chem. 478 (2025) 143597-143597. https://doi.org/10.1016/j.foodchem.2025.143597.
